# Accumulation of formaldehyde causes motor deficits in an in vivo model of hindlimb unloading

**DOI:** 10.1038/s42003-021-02448-9

**Published:** 2021-08-19

**Authors:** Dandan Yao, Qingyuan He, Shangying Bai, Hang Zhao, Jun Yang, Dehua Cui, Yan Yu, Xuechao Fei, Yufei Mei, Ye Cheng, Shi Yan, Nayan Huang, Yalan Di, Xianjie Cai, Rui Wang, Yajuan Gao, Fangxiao Cheng, Shengjie Zhao, Xu Yang, Xiang Cai, Hongbin Han, Jihui Lyu, Zhiqian Tong

**Affiliations:** 1 Beijing Institute of Brain Disorders, Laboratory of Brain Disorders, Ministry of Science and Technology, Collaborative Innovation Center for Brain Disorders, Capital Medical University, Beijing, China; 2 Center for Cognitive Disorders, Beijing Geriatric Hospital, Beijing, China; 3 Institute of Aging, Key Laboratory of Alzheimer’s Disease of Zhejiang Province, School of Mental Health, Wenzhou Medical University, Wenzhou, China; 4 Department of Radiology, Peking University Third Hospital, Beijing, Key Laboratory of Magnetic Resonance Imaging Equipment and Technique, Beijing, China; 5 Chinese institute of Rehabilitation Science, China Rehabilitation Research Center, Beijing Key Laboratory of Neural Injury and Rehabilitation, Beijing, China; 6 Section of Environmental Biomedicine, Hubei Key Laboratory of Genetic Regulation and Integrative Biology, College of Life Sciences, Central China Normal University, Wuhan, China

**Keywords:** Movement disorders, Cerebellum

## Abstract

During duration spaceflight, or after their return to earth, astronauts have often suffered from gait instability and cerebellar ataxia. Here, we use a mouse model of hindlimb unloading (HU) to explore a mechanism of how reduced hindlimb burden may contribute to motor deficits. The results showed that these mice which have experienced HU for 2 weeks exhibit a rapid accumulation of formaldehyde in the gastrocnemius muscle and fastigial nucleus of cerebellum. The activation of semicarbazide-sensitive amine oxidase and sarcosine dehydrogenase induced by HU-stress contributed to formaldehyde generation and loss of the abilities to maintain balance and coordinate motor activities. Further, knockout of formaldehyde dehydrogenase (FDH^-/-^) in mice caused formaldehyde accumulation in the muscle and cerebellum that was associated with motor deficits. Remarkably, formaldehyde injection into the gastrocnemius muscle led to gait instability; especially, microinfusion of formaldehyde into the fastigial nucleus directly induced the same symptoms as HU-induced acute ataxia. Hence, excessive formaldehyde damages motor functions of the muscle and cerebellum.

## Abstract (web summary)

Dandan Yao et al. demonstrated that accumulation of formaldehyde in the cerebellum and skeletal muscle contributed toward motor deficits in a mouse model of hindlimb unloading. Their results collectively suggest that scavenging formaldehyde may be a useful treatment to limit motor dysfunction following limb offloading conditions.

## Introduction

An important question in aerospace medicine or space medico-engineering is how to protect an astronaut’s health^1^. Disconcertingly, astronauts exposed to microgravity and space travel have exhibited several neurologic changes, including acute ataxia, postural disturbances, perceptual illusions, neuromuscular weakness, and fatigue^2,3^. Even several weeks after they return to earth, astronauts are still relearning how to walk^4–6^. Actually, the results of magnetic resonance imaging (MRI) after long space flight showed that the structure of the cerebellum had been altered in the astronauts^7^. Similarly, anatomical observations revealed that the cerebellums of rats were damaged during spaceflight^8^. In a rat model of hindlimb unloading (HU, a model of partially simulated microgravity stress), although muscle atrophy in the hind limbs had recovered after a 2-week recovery period, the locomotor deficits were not reversed^9,10^. These data strongly suggest that a cerebellum impaired by microgravity may be a direct and critical factor in the cerebellar ataxia experienced by astronauts.

The fastigial nucleus (FN) is the phylogenetically oldest nucleus in the cerebellum, a classical subcortical motor coordinator^11^. Clinical investigations have found that an FN lesion or dysfunction results in motor deficits, including cerebellar ataxias, and nonmotor symptoms in humans^12^. Under microgravity conditions, the expression of semicarbazide-sensitive amine oxidase (SSAO, a formaldehyde-generating enzyme) was elevated about 2-fold in a system of SSAO-transformed *Escherichia coli* BL21^13^. Notably, exogenous formaldehyde or methanol (MeOH, a precursor of formaldehyde^14^) poisoning also induced severe cerebellar ataxias in mice^15^ and humans^16^. Recent studies revealed that the accumulated formaldehyde induces muscle atrophy and osteoporosis in the mice with double-knockout of alcohol dehydrogenase-5 (ADH5) and aldehyde dehydrogenase-2 (ALDH2) (two enzyme of clearance formaldehyde)^17,18^. Thus, an abnormal elevation in the levels of systemic formaldehyde may contribute to motor deficits of astronauts.

In this study, we investigated whether HU-stress induces formaldehyde accumulation in the muscle and cerebellum and then leads to motor disorders including: gait instability and cerebellar ataxia. The findings showed that HU-stress not only promotes formaldehyde generation by activating these formaldehyde-generating enzymes (such as: mitochondrial sarcosine dehydrogenase, SARDH, and blood/vascular SSAO), but also inhibits formaldehyde degradation by reducing the expression and activity of formaldehyde dehydrogenase (FDH, also named ADH5 or Aldh1l1). Interestingly, the combination with 630-nm red light and nano-packed coenzyme Q10 has a better therapeutic effect in degrading systemic formaldehyde and restoring motor functions than single treatment.

## Results

### HU stress-induced systemic formaldehyde accumulation and motor deficits

**Fig. 1:**
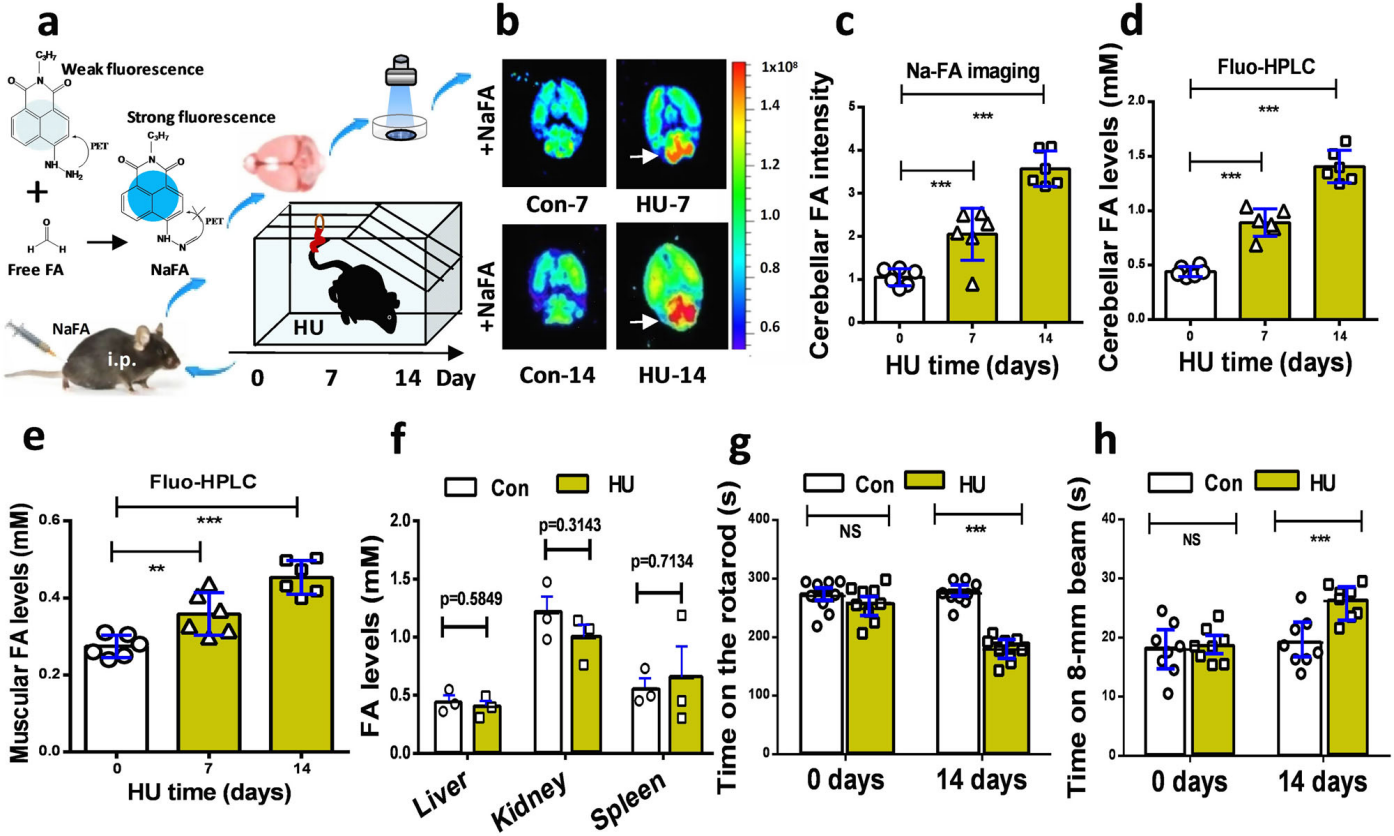
Mouse model of hindlimb unloading exhibited cerebellar formaldehyde accumulation and motor deficits. **a** Schematic overview of a mouse model established by subjecting the hind limbs to unloading for 2 weeks. i.p. intraperitoneal injection, FA formaldehyde, HU hindlimb unloading, NaFA a free FA fluorescence probe. **b** Brain formaldehyde imaged by an in-vitro small animal system with NaFA probe (*λ*_ex\em_ = 440/550 nm). **c** Cerebellar formaldehyde quantified by Na-FA fluorescence intensity (*n* = 6). FA formaldehyde. **d** Cerebellar formaldehyde detected by Fluo-HPLC. (*n* = 6). Fluo-HPLC high-performance liquid chromatography with a fluorescence detector. **e** Muscular FA detected by Fluo-HPLC (*n* = 6). **f** FA levels in the liver, kidney, and spleen examined by FA kits (*n* = 6). **g**, **h** Motor functions evaluated by the beam walking test (*n* = 8). Error bars show the mean ± SEM; NS no statistical significance; ***p < 0.001.

To elucidate the relationship between cerebellar formaldehyde levels and motion functions under weightless conditions, we first made a mouse model of HU stress by subjecting the hind limbs to unloading for 2 consecutive weeks as described previously^10,19,20^, and then examined the formaldehyde concentrations in the brain and assessed motor behaviors. Using an in vivo small animal imaging system with the free formaldehyde fluorescence probe NaFA (*λ*_ex/em_ = 440/550 nm)^21,22^ (Fig. 1a), we found that HU-stress induced a time-dependent elevation in the fluorescence intensity due to cerebellar formaldehyde (values on day 7 vs. day 0: *n* = 6, *t* = 7.827, df = 10, *p* < 0.001; values on day 14 vs. day 0: *n* = 6, *t* = 11.43, df = 10, *p* < 0.001; one-way ANOVA). The darker color of red means the stronger fluorescence of formaldehyde (Fig. 1b, c). To quantify the concentrations of formaldehyde in the cerebellum, we used the classical method of high-performance liquid chromatography with a fluorescence detector (Fluo-HPLC)^23,24^. The results showed that cerebellar formaldehyde levels in the model group on day 14 were elevated 3-fold compared to the control group (values on day 7 vs. day 0: *n* = 6, *t* = 7.537, df = 10, *p* < 0.001; values on day 14 vs. day 0: *n* = 6, *t* = 20.77, df = 10, *p* < 0.001; one-way ANOVA) (Fig. 1d). Meanwhile, we also observed a time-dependent elevation in the levels of formaldehyde in the gastrocnemius muscle of the mice of the HU group (0 day: 0.2741 ± 0.0112; 7 days: 0.3583 ± 0.0223; 14 days: 0.4534 ± 0.0179. *t* = 8.332, df = 10, *p* < 0.001; one-way ANOVA) (Fig. 1e). However, there was no significant difference in the levels of FA in the liver, kidney and spleen between these HU mice and wild-type mice (*p* > 0.05) (Fig. 1f).

To address whether HU stress leads to motor deficits, the two methods of the accelerated rotarod test and beam walking test were used to assess the abilities of the mice to maintain balance/motor coordination and sensorimotor functions, respectively. The results showed that the staying times on the rotarod in the HU group on day 14 were markedly shorter than those of the control group (Con) (values of HU vs. Con on day 0: *n* = 9, *t* = 0.5008, df = 16, *p* = 0.6233; values of HU vs. Con on day 14 vs. day 0: *n* = 9, *t* = 6.622, df = 16, *p* < 0.001; unpaired *t*-test) (Fig. 1g and Supplementary Fig. 1a). Meanwhile, times required for crossing the 8-mm beam were longer in the model group than in the control group (values of HU vs. Con on day 0: *n* = 8, *t* = 0.5846, df = 14, *p* = 0.5846; values of HU vs. Con on day 14 vs. day 0: *n* = 8, *t* = 5.282, df = 14, *p* < 0.001; unpaired *t*-test) (Fig. 1h and Supplementary Fig. 1b). These data indicated that HU stress indeed caused motor deficits, and suggested that formaldehyde accumulation in both the muscle and cerebellum may be closely related to motion disorders.

### HU elicited cerebellar formaldehyde accumulation by disturbing its metabolism

**Fig. 2:**
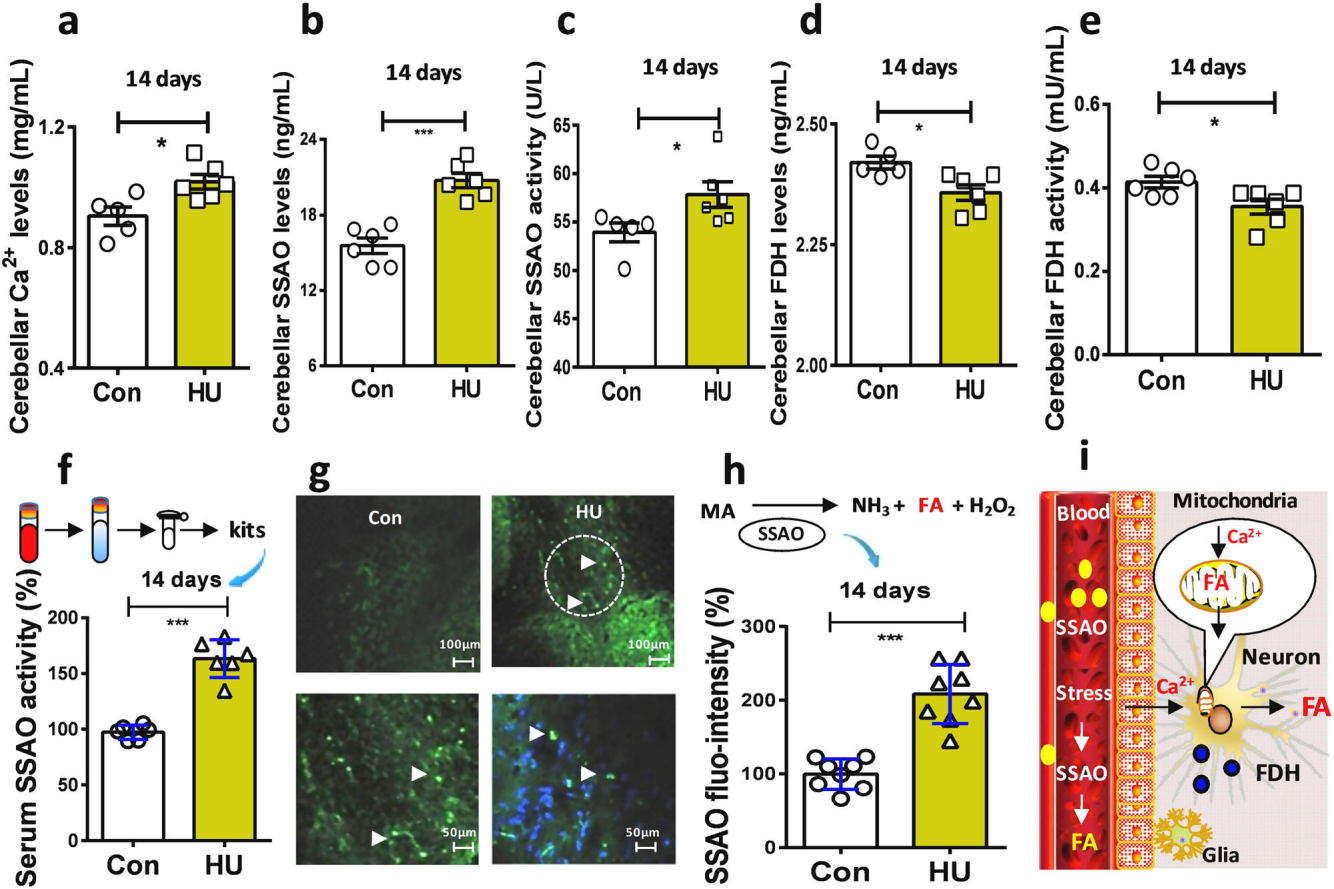
Hindlimb unloading caused formaldehyde accumulation by affecting intracellular Ca2+ and FA metabolism in vivo. **a** HU-induced the elevation in brain Ca^2+^ levels. (*n* = 6). HU hindlimb unloading. **b**, **c** Changes in the contents and activities of SSAO in the cerebellum of HU mice. (*n* = 6). SSAO semicarbazide-sensitive amine oxidase. **d**, **e** Changes in the contents and activities of brain FDH. (*n* = 6). FDH formaldehyde dehydrogenase. **f** Blood SSAO activity detected using a mouse SSAO kit. (*n* = 6). **g** SSAO (green) located in the cerebellum. DAPI (blue, a dye for nuclear). **h** Statistical analysis of cerebellar SSAO expression (*n* = 8). **i** Model of stress-induced FA generation. FA formaldehyde, FDH formaldehyde dehydrogenase, SSAO semicarbazide-sensitive amine oxidase. Error bars show the mean ± SEM; *p < 0.05, ***p < 0.001.

Our above data showed that formaldehyde levels were markedly elevated in the brains. This suggests that weightiness activates neurons associated with disturbing formaldehyde metabolism in these HU model mice. Intracellular Ca^2+^ signaling is essential for neuronal activity and formaldehyde generation^25^. We examined brain Ca^2+^ contents with the quantichrom calcium assay kit. The results showed that there was a marked elevation in the levels of brain Ca^2+^ in the HU group than control group (*t* = 2.987, df = 9, *p* = 0.0153; unpaired *t*-test) (Fig. 2a). Next, we investigated the changes in formaldehyde-metabolic enzymes. SSAO distributed in the smooth muscles, vascular walls, and blood is sensitive to stress and produces formaldehyde^26,27^, which has been found to be sensitive to microgravity^13^. In addition, FDH is a specifical formaldehyde-degrading enzyme^28^. Our results showed that the expression and activity of SSAO were markedly elevated in the HU group than control group (expression: *t* = 6.167, df = 10, *p* < 0.001; activity: *t* = 2.293, df = 9, *p* = 0.0476; unpaired *t*-test) (Fig. 2b, c). However, both the expression and activity of FDH in the HU group were decline compared with control group (expression: *t* = 2.892, df = 9, *p* = 0.0178; activity: *t* = 2.588, df = 9, *p* = 0.0270; unpaired *t*-test) (Fig. 2d, e),

Since SSAO is also presented in the blood^26,27^, we examined the expression and activity of serum SSAO activity in these groups of mice. The results showed that SSAO activity in the HU group was 1.5-fold higher than that in the control group after 14 days of HU (*n* = 6, *t* = 8.962, df = 10, *p* < 0.0001; unpaired *t*-test) (Fig. 2f). Meanwhile, the results of fluorescence immunochemistry revealed that SSAO (green) was mainly expressed in the vascular walls and not distributed in the neuronal cells (blue, nuclei stained by DAPI) (Fig. 2g). In particular, SSAO expression was 2-fold higher in the HU model group compared to the control group (*n* = 8, *t* = 6.819, df = 14, *p* < 0.0001; unpaired *t*-test) (Fig. 2h). Thus, both the activation of SSAO and inhibition of FDH lead to formaldehyde accumulation in the brains of these HU mice (Fig. 2i).

### Microgravity induced formaldehyde generation in a rotary culture system

**Fig. 3:**
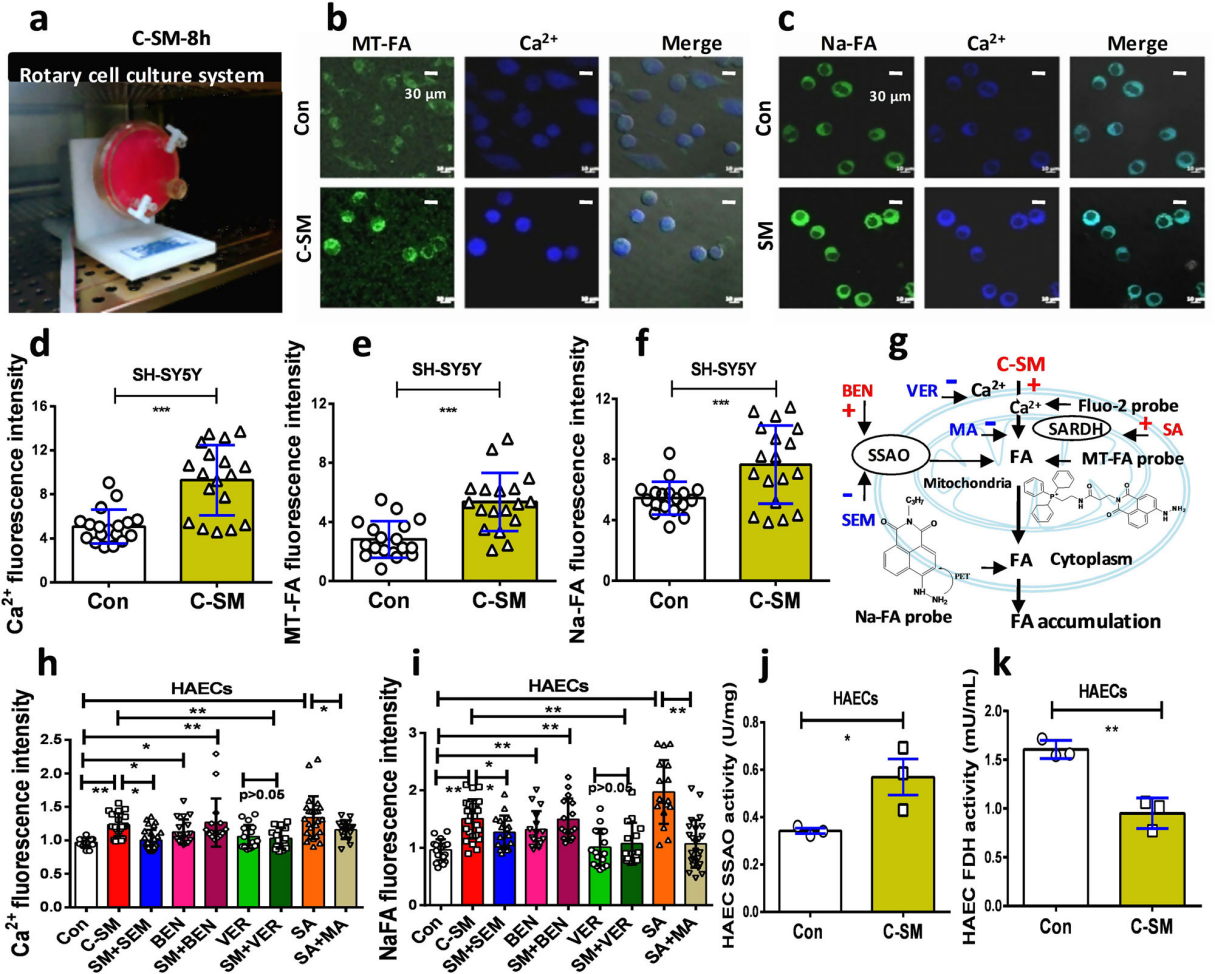
Microgravity promoted formaldehyde generation by affecting intracellular Ca2+ and FA metabolism in vitro. **a** Simulated microgravity model of human SH-SY5Y cells in a rotary cell culture system. C-SM: simulated microgravity in the cultured cells. **b**, **c** Mitochondrial or cytoplasmic formaldehyde and intracellular Ca^2+^ detected by confocal imaging. Scale bar = 30 μm. **d**–**f** Statistical analysis of cellular Ca^2+^ influx, mitochondrial formaldehyde, and cytoplasmic formaldehyde (*n* = 18). 8 h: 8 h. **g** Cell model of the effects of the different medicines on FA metabolism and intracellular Ca^2+^ levels. FA formaldehyde, SSAO semicarbazide-sensitive amine oxidase, SARDH sarcosine dehydrogenase, BEN benzylamine, MA methoxyacetic acid, SA sarcosine, SEM semicarbazide, VER verapamil. **h**, **i** Changes in the levels of intracellular Ca^2+^ and FA in the cultured HAECs after different medicines treatments, respectively. HAECs human aortic endothelial cells. **j**, **k** Changes in the activity of SSAO and FDH in the cultured HAECs. (Repeated three times). Error bars show the mean ± SEM; *p < 0.05, **p < 0.01, ***p < 0.001.

Next, we investigated whether microgravity induces formaldehyde generation in cultured human SH-SY5Y (SARDH expressed in these cells) and HAECs (both SARDH and SSAO expressed in these cells) in a rotary culture system (Fig. 3a and Supplementary Movie 1). After the cultured cells with 8 h of rotation under conditions of simulated microgravity (C-SM), we found that formaldehyde had been rapidly generated in the mitochondria of the cultured neurons and was associated with a marked intracellular Ca^2+^ influx (Fig. 3b, c), which was detected by a mitochondrial formaldehyde-sensitive fluorescence probe (*λ*_ex/em_ = 440/550 nm)^29^ and a Ca^2+^-sensitive probe Fura-2 AM, respectively^30^. Meanwhile, we observed the effects of microgravity on cytoplasmic formaldehyde as quantified by a free formaldehyde fluorescence probe^22^. Our results indicated that the cultured neuronal cells exhibited marked elevations in the levels of intracellular Ca^2+^ (*n* = 18, *t* = 5.027, df = 34, *p* < 0.0001; unpaired *t*-test) (Fig. 3d), mitochondrial formaldehyde (*n* = 18, *t* = 4.605, df = 34, *p* < 0.0001; unpaired *t*-test) (Fig. 3e), and cytoplasmic formaldehyde (*n* = 18, *t* = 3.344, df = 34, *p* < 0.0001; unpaired *t*-test) (Fig. 3f). Thus, microgravity caused the generation and release of formaldehyde from the cerebellar neurons (Fig. 3g).

Next, we investigated whether HU-induced Ca^2+^ influx is dependent on Ca^2+^-channel by using its blocker-verapamil (VER), identified whether the disturbance of SSAO by semicarbazide (SEM, an inhibitor of SSAO) and benzylamine (BEN, a substrate of SSAO) affects formaldehyde metabolism, and examined the effects of methoxyacetic acid (MA, an inhibitor of SARDH) and sarcosine (SA, a substrate of SARDH). The results showed that HU induced a marked elevation in the levels of the intracellular Ca^2+^ influx (*t* = 5.765, df = 33, *p* < 0.001; one-way ANOVA), while VER (a blocker of Ca^2+^-channel) could alleviate this change. Formaldehyde can promote intracellular Ca^2+^ influx in the cultured neurons and primary keratinocytes as well as in non-neuronal cell lines^25,31^. Unsurprisingly, BEN and SA enhanced Ca^2+^ influx, respectively (BEN: *t* = 3.109, df = 31, *p* < 0.01; SA: *t* = 4.377, df = 38, *p* < 0.001, one-way ANOVA) (Fig. 3h and Supplementary Fig. 2). Similarly, these medicines made the corresponding trends in formaldehyde generation detected by NaFA probe (Fig. 3i and Supplementary Fig. 2). As expected, microgravity induced an increased expression in SSAO associated with a decline in the contents of FDH in the cultured HAECs (SSAO: *t* = 2.951, df = 4, *p* = 0.0419; FDH: *t* = 6.237, df = 4, *p* = 0.0034; unpaired *t*-test) (Fig. 3j, k). Taken together, microgravity indeed disturbs formaldehyde metabolism and results in formaldehyde accumulation in both the model cells and mice.

### Knockout of *FDH* led to systemic formaldehyde accumulation and motor deficits

**Fig. 4:**
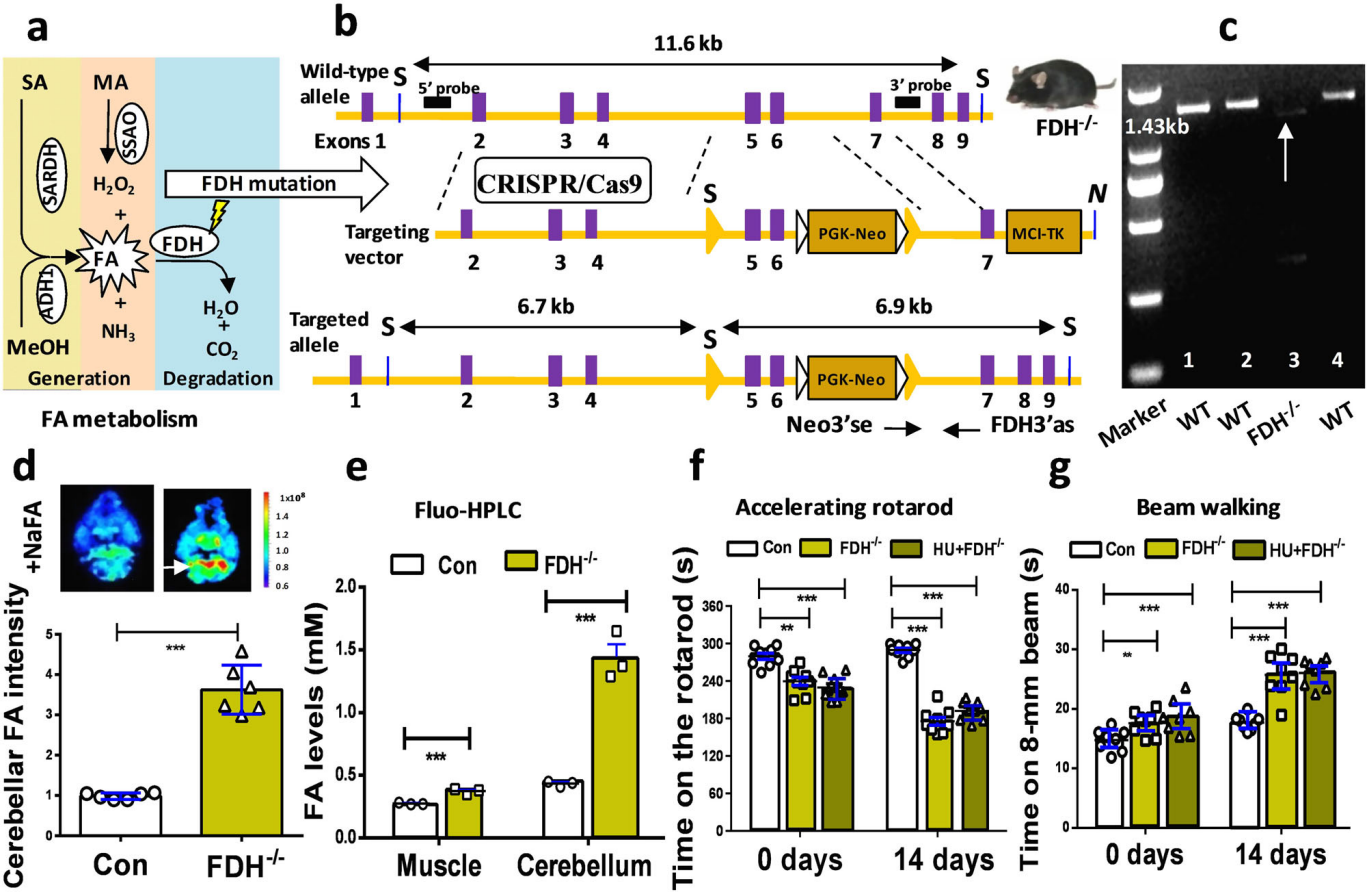
Knockout of FDH induced the accumulation of systemic formaldehyde and motor deficits. **a** Metabolic pathways of formaldehyde including: formaldehyde generation by sarcosine dehydrogenase (SARDH), semicarbazide-sensitive amine oxidase (SSAO), and alcohol dehydrogenase 1 (ADH1); and degradation by formaldehyde dehydrogenase (FDH). SA sarcosine, MA methylamine, MeOH methanol, FA formaldehyde. **b** Technology roadmap of making *FDH*^−/−^ mice by using the CRISPR/Cas9 method to delete the exons- 5 and 6. **c** *FDH*^−/−^ mice identified by RT-PCR (target band, 1.43 kb). WT wild-type mice, *FDH*^−/−^ the *FDH*-knockout mice. **d** Cerebellar formaldehyde quantified by an in vitro small animal imaging system (*n* = 6). **e** Formaldehyde levels in the muscle and cerebellum detected by Fluo-HPLC (*n* = 3). Fluo-HPLC high-performance liquid chromatography with a fluorescence detector. **f** Motor behaviors assessed by the accelerating rotarod test (*n* = 9). HU hindlimb unloading. **g** Motor functions evaluated by the beam walking test (*n* = 8). Error bars show the mean ± SEM; **p < 0.01; ***p < 0.001.

The above data indicated that HU caused motor deficits associated with formaldehyde accumulation in the muscle and cerebellum. Unexpectedly, exogenous formaldehyde or methanol (a precursor of formaldehyde) has been shown to induce cerebellar ataxias in rats^15^ and humans^16^. These data suggest that excessive formaldehyde in the cerebellum may be a direct trigger of acute cerebellar ataxia in the HU mouse model. Endogenous formaldehyde is derived from multiple metabolic pathways, including sarcosine (SA) degraded by SARDH^25^, methylamine (MA) demethylated by SSAO^32^, and methanol (MeOH) translated by alcohol dehydrogenase 1 (ADH1)^33^, while it is mainly degraded by formaldehyde dehydrogenase (FDH)^34^ (Fig. 4a). To establish the direct links between formaldehyde accumulation and cerebellar impairment, we made a formaldehyde-sufficient mouse model by using the CRISPR/Cas9 technique to knockout the *FDH* gene (Fig. 4b and Supplementary Fig. 3–7). DNA of 1.43 kb and a protein of 40 kDa corresponding to FDH in these *FDH*^−/−^ mice were identified by the methods of reverse transcription-polymerase chain reaction (RT-PCR) and western blotting, respectively (Fig. 4c and Supplementary Figs. 20 and 8a, b).

The cerebellar formaldehyde levels were quantified using an in vivo small animal imaging system with an NaFA probe^22^ and Fluo-HPLC^23^, respectively. We found that the formaldehyde fluorescence intensity in *FDH*^−/−^ mice was elevated 3-fold compared to that in control mice (*n* = 6, *t* = 17.82, df = 10, *p* < 0.0001; unpaired *t*-test) (Fig. 4d). Using the Fluo-HPLC method, we found that muscular formaldehyde levels in *FDH*^−/−^ mice were higher than those in control mice (*n* = 3, *t* = 4.093, df = 4, *p* < 0.0001; unpaired *t-*test), and associated with higher levels of cerebellar formaldehyde in the former than in the latter (*n* = 3, *t* = 18.426, df = 4, *p* < 0.0001; unpaired *t*-test) (Fig. 4e). These data indicated that *FDH* knockout led to formaldehyde accumulation in the muscle and cerebellum.

Next, we assessed the motor behaviors by the two methods of the accelerating rotarod test and beam walking test. The results showed that *FDH*^−/−^ mice and HU-treated *FDH*^−/−^ mice on days 0 and 14 remained for shorter times on the rotarod than control mice (values on days 0, Con: 281.5 ± 5.340; HU: 239.6 ± 6.464; HU + *FDH*^−/−^: 229.5 ± 6.345. Values on days 14, Con: 289.5 ± 3.410; HU: 175.7 ± 6.404; HU + *FDH*^−/−^: 192.4 ± 5.041; *p* < 0.001; unpaired *t*-test) (Fig. 4f). In addition, times for crossing the 8-mm beam in *FDH*^−/−^ mice and HU-treated *FDH*^−/−^ mice on days 0 and 14 were longer than those in control mice (values on days 0, Con: 14.76 ± 0.6635; HU: 17.58 ± 0.7242; HU + *FDH*^−/−^: 18.67 ± 1.014. Values on days 14, Con: 17.68 ± 0.4452; HU: 25.73 ± 1.260; HU + *FDH*^−/−^: 26.05 ± 0.8180; *p* < 0.001; unpaired *t*-test) (Fig. 4g). The above data demonstrated that *FDH*^−/−^ knockout indeed led to systemic formaldehyde accumulation, and to deficiencies in the abilities to maintain balance/motor coordination and in the sensorimotor functions.

### Formaldehyde injection into the gastrocnemius muscle induces gait instability

**Fig. 5:**
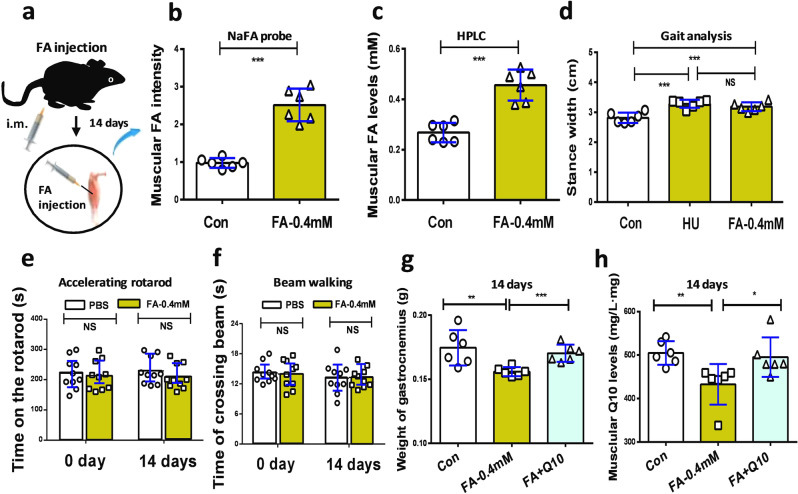
Injection of formaldehyde into the gastrocnemius muscle led to gait instability in healthy wild-type mice. **a** Formaldehyde at 0.4 mM was injected into the muscle of hindlimb for 14 consecutive days. i.m. intramuscular injection. **b** Muscular formaldehyde levels quantified by NaFA probe (*n* = 6). **c** Formaldehyde levels in the gastrocnemius detected by HPLC (*n* = 6). HPLC high-performance liquid chromatography. **d** Gait analysis of the stance width in Con, HU model, and FA-injected mice. Con: the wild-type mice; HU: hindlimb unloading; FA: formaldehyde (*n* = 6). **e** Motor behaviors assessed by the accelerating rotarod test (*n* = 6). **f** Motor functions evaluated by the beam walking test (*n* = 10). **g** Weights of gastrocnemius (*n* = 6). **h** Muscular Q10 levels detected by Q10 kits (*n* = 6). Error bars show the mean ± S.E.M; NS no statistical significance; *p < 0.05; **p < 0.01; ***p < 0.001.

Although *FDH*^−/−^ mice also exhibited motor deficits, this kind of systemic *FDH* knockout not only caused formaldehyde accumulation in the brain but also induced it to be stored in the peripheral organs including muscles (Fig. 4e), which affect motor behaviors^10^. To address the roles of the accumulated muscular formaldehyde in the motor behaviors in wild-type mice, we directly injected formaldehyde into the gastrocnemius muscle of the hindlimb of wild-type mice for 14 consecutive days (Fig. 5a). The results of Fluo-HPLC investigations showed that formaldehyde concentrations were elevated in the gastrocnemius muscle (fluorescence values in FA-0.4 mM vs. Con: *n* = 6, *t* = 13.97, df = 10, *p* < 0.0001; formaldehyde levels in FA-0.4 mM vs. Con: *n* = 6, *t* = 21.27, df = 10, *p* < 0.0001; unpaired *t*-test) (Fig. 5b, c).

Muscle injury has been found to induce abnormal gaits in mice^35,36^. As expected, these mice with intramuscular injection of formaldehyde (i.m.) exhibited a longer stance width (FA-0.4 mM vs. Con: 3.283 ± 0.054, 2.815 ± 0.0715; *t* = 5.222, df = 10, *p* = 0.0004; one-way ANOVA) (Fig. 5d and Supplementary Movie 2), and shorter stride length, but no difference in stride frequency than controls (Supplementary Fig. 9). Meanwhile, we compared the changes in stance width of the group of FA-0.4 mM with the HU group, and found that there was no difference between these two groups (stance width of FA-0.4 mM vs. HU: 3.283 ± 0.054,3.285 ± 0.0614; *t* = 1.201, df = 10, *p* = 0.2574; one-way ANOVA) (Fig. 5d). Thus, the accumulated formaldehyde in the muscle resulted in gait abnormality.

Further, the results of motor behavior assessments showed that times on the accelerating rotarod were not diffident in the formaldehyde-injected mice compared to the control mice (values of FA-0.4 mM vs. Con on day 14: *n* = 10, *t* = 1.061, df = 18, *p* = 0.3026; unpaired *t*-test) (Fig. 5e). Moreover, the times required to cross the 8-mm beam in the formaldehyde-injected mice were not diffident than those in the control mice (values of FA-0.4 mM vs. Con on day 14: *n* = 10, *t* = 0.1429, df = 18, *p* = 0.8812; unpaired *t*-test) (Fig. 5f). Thus, excessive formaldehyde in the muscle mainly induced gait instability but not motor functions.

Previous studies showed that supplementation of coenzyme Q10 contributes to the recovery of muscular atrophy^37,38^. Thus, we explored whether Q10 rescues formaldehyde-induced muscle injure. After intragastric administration of Nano-Q10 (30-nm coenzyme Q10, diameter: 30–40 nm, which can enhance the water solubility and liposolubility of the non-water-soluble Q10) for 14 consecutive days, Q10 could improve the weights of the gastrocnemius muscle in the formaldehyde-injected mice (Con vs. FA-0.4 mM (mg): 174.5 ± 5.638; 155.9 ± 1.434, *p* = 0.0096; FA-0.4 mM vs. FA plus Q10 (mg): 155.9 ± 1.434, 170.3 ± 2.733, *p* = 0.0009; one-way ANOVA) (Fig. 5g). It also increased the muscular Q10 contents (Con vs. FA-0.4 mM: 504.8 ± 11.13; 432.7 ± 19.05, *p* = 0.0085; FA-0.4 mM vs. FA plus Q10: 432.7 ± 19.05, 495.3 ± 18.57, *p* = 0.0405; one-way ANOVA) (Fig. 5h). Hence, Nano-Q10 could rescue formaldehyde-induced muscle atrophy by restoring muscular Q10 levels.

### Formaldehyde-infused into the cerebellum directly causes ataxia

**Fig. 6:**
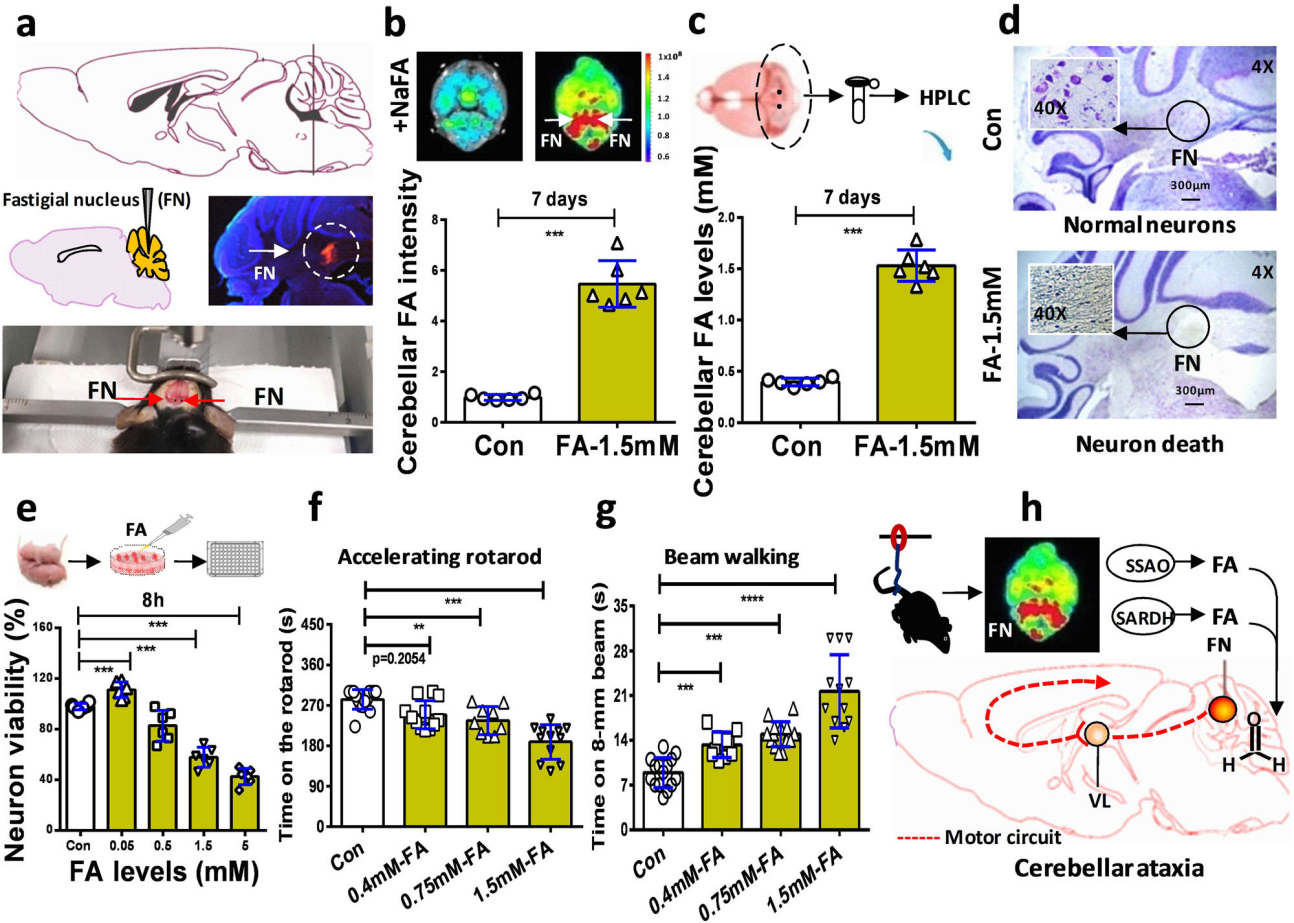
Formaldehyde-infused into the fastigial nucleus promoted formaldehyde overload and motor deficits. **a** Location, microinfusion, and identification of the bilateral fastigial nucleus injected with formaldehyde. FN fastigial nucleus (location identified by using red fluorescent probe-Dil to mark cell membrane and blue DAPI to stain nucleus). **b** Cerebellar formaldehyde quantified by an in-vitro small animal imaging system (*n* = 6). FA-1.5 mM: the group of mice microinfused with 1.5 mM formaldehyde; Con PBS-injected group of mice, FA formaldehyde. **c** Formaldehyde levels in the cerebellum detected by HPLC (*n* = 6). HPLC high-performance liquid chromatography. **d** Cellular morphology of the cerebellar neurons detected by using Nissl staining solutions (*n* = 3). **e** Cell toxicity of formaldehyde detected by using a CCK-8 kit (*n* = 6). **f** Motor behaviors assessed in FA-injected mice by the accelerating rotarod test (*n* = 10). **g** Motor functions in FA-injected mice evaluated by the beam walking test (*n* = 10). **h** The model of FN impairments results in cerebellar ataxia induced by HU-derived formaldehyde. VL ventrolateral nucleus of the thalamus. Error bars show the mean ± SEM; **p < 0.01; ***p < 0.001.

To rule out the effects of formaldehyde on the motor functions of the muscle, we directly infused formaldehyde into the bilateral FN (marked red) of the cerebellums of healthy adult male wild-type mice for 7 consecutive days according to the mouse brain in stereotaxic coordinates^39^ (Fig. 6a and Supplementary Fig. 10–12). The results of both the in vivo small animal imaging system and Fluo-HPLC investigations showed that formaldehyde concentrations were elevated in the cerebellums (fluorescence values in FA-1.5 mM vs. Con: *n* = 6, *t* = 61.81, df = 10, *p* < 0.0001; formaldehyde levels in FA-1.5 mM vs. Con: *n* = 6, *t* = 25.71, df = 10, *p* < 0.0001; unpaired *t*-test) (Fig. 6b, c).

Then, we examined the neurotoxicity of excessive formaldehyde in the cerebellar neurons in vivo and in vitro. After formaldehyde infusion into the FN for 7 days, we examined the death of cerebellar neurons by staining with Nissl solutions, and found that a formaldehyde concentration of 1.5 mM led to a marked decrease in the numbers of cerebellar neurons in formaldehyde-injected wild-type mice (Fig. 6d and Supplementary Figs. 11–12). Further, the results in formaldehyde-treated cultured cerebellar neurons showed that incubation with formaldehyde at concentrations over 0.05 mM led to a dose-dependent decline in the viability of the cultured neurons examined using the Cell Counting Kit-8 (CCK-8 kit) (*n* = 6, *t* = 18.63, df = 10, *p* < 0.0001; one-way ANOVA) (Fig. 6e). The data from these in vitro and in vivo experiments indicated that excessive formaldehyde directly induced the death of cerebellar neurons.

Further, we investigated whether formaldehyde injection into the FN directly results in cerebellar ataxia in healthy adult wild-type mice. As expected, after formaldehyde was injected into the FN for 7 consecutive days, these mice exhibited severe symptoms of acute cerebellar ataxia, for example, difficulty maintaining normal upright posture, balance, coordinated walking, and running, associated with unsteady gait, staggering, tripping, and falling (Supplementary Movie 3). Notably, microinfusion of formaldehyde at 0.4, 0.75, and 1.5 mM induced a dose-dependent decline in the times on the accelerating rotarod in these healthy wild-time mice (Con: 283 ± 5.714, 0.4 mM-FA: 249.3 ± 9.102; 0.75 mM-FA: 236.2 ± 10.43; 1.5 mM-FA: 188.7 ± 11.06; *t* = 3.035, df = 19, *p* < 0.01; one-way ANOVA) (Fig. 6f and Supplementary Movies 4 and 5). Similarly, there was a dose-dependent increase in times on 8-mm beam The results of the beam walking test also showed that the times required to cross the 8-mm beam (Con: 8.933 ± 0.5973; 0.4 mM-FA: 13.28 ± 0.6262; 0.75 mM-FA: 14.93 ± 1.653; 1.5 mM-FA: 21.67 ± 1.653; *t* = 4.274, df = 25, *p* < 0.001; one-way ANOVA) (Fig. 6g and Supplementary Movies 6–9). The above data demonstrated that excessive formaldehyde in the FN of the cerebellum directly induced the death of cerebellar neurons, and impaired motor circuit from FN to cortex;^11^ subsequently, it led to loss of the abilities to maintain balance/motor coordination and sensorimotor functions (Fig. 6h).

### Scavenging systemic formaldehyde reversed HU-induced motor deficits

**Fig. 7:**
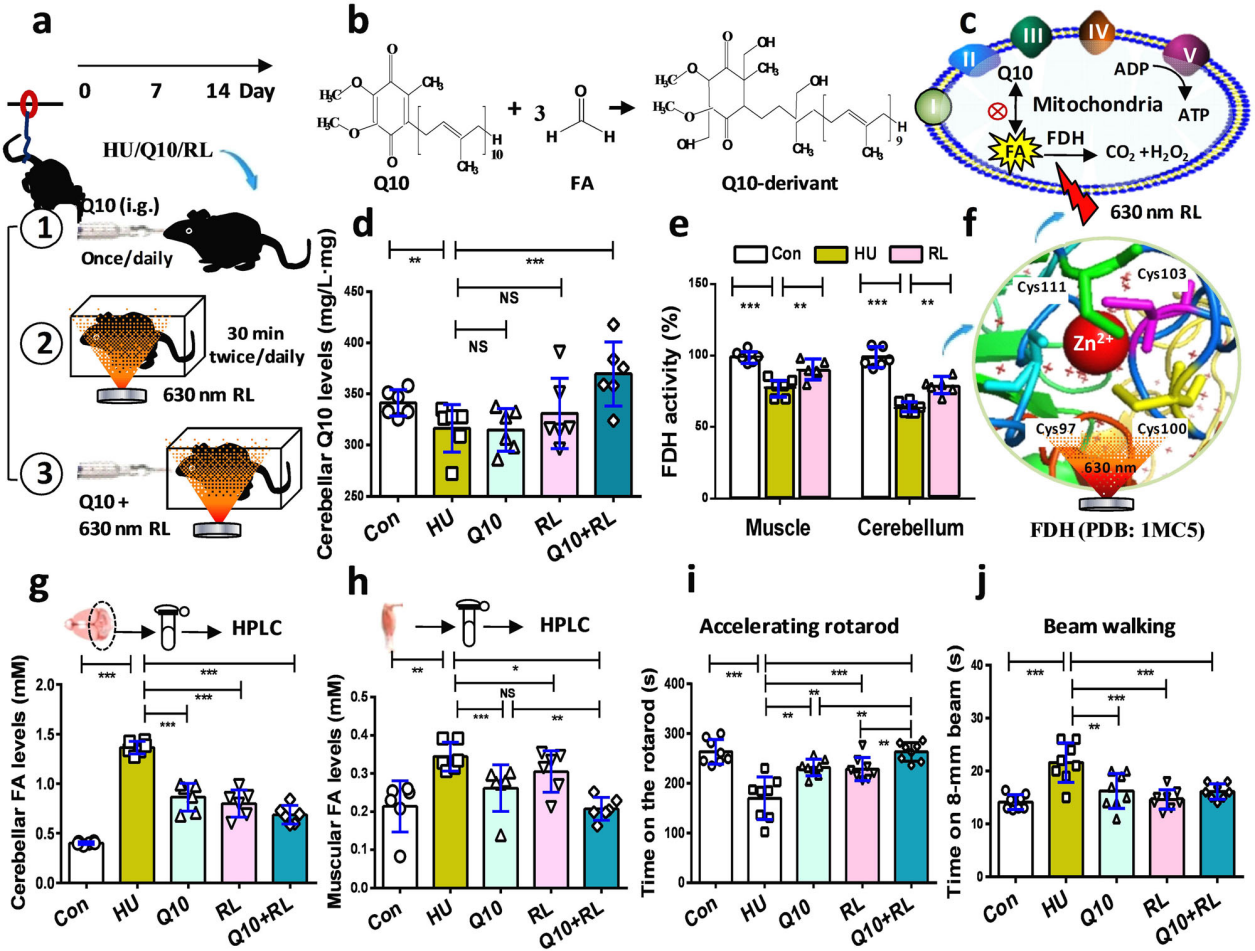
Reduction of systemic formaldehyde rescued motor functions in the mice of hindlimb unloading. **a** The experimental flow. HU: mice of hindlimb unloading; Q10: HU mice intragastrically administered (i.g.) 30-nm coenzyme Q10; RL: HU mice illuminated with 630-nm red light. **b** The spontaneous chemical reaction between Q10 and formaldehyde. FA formaldehyde. **c** Metabolism of formaldehyde and coenzyme Q10 in the mitochondria. **d** Coenzyme Q10 levels quantified by the ECA-fluorescence method (*n* = 6). **e** FDH activity detected using an FDH kit (*n* = 6). FDH formaldehyde dehydrogenase. **f** Schematic diagram of the effects of and the activation of the Cys97 and Cys100 residues in FDH by 630-nm red light (PDB: 1MC5). Cys: cysteine; Cys111 (green); Cys103 (purple); Cys97 (dusty blue); Cys100 (yellow). **g**, **h** Formaldehyde levels quantified by HPLC (*n* = 6). HPLC high-performance liquid chromatography. **i**, **j** Motor functions assessed by accelerating rotarod and beam walking tests, respectively (*n* = 8). Error bars show the mean ± S.E.M; **p < 0.01; ***p < 0.001.

If accumulations of formaldehyde in the muscle and cerebellum lead to muscular atrophy and cerebellar ataxia, scavenging formaldehyde should improve motor functions. To reduce systemic formaldehyde, the mice in the HU group were intragastrically administered Nano-Q10 (Q10 group) for 14 consecutive days, illuminated with 630-nm red light (RL group)^40^, or treated with Q10 plus RL (Q10 + RL group) (Fig. 7a and Supplementary Fig. 13). In patients with Alzheimer’s disease, the concentrations of Q10 and formaldehyde varied inversely^41,42^, suggesting that these two compounds undergo a spontaneous chemical reaction. In this study, we found that one molecule of Q10 could scavenge three molecules of formaldehyde, as identified by gas chromatography-tandem mass spectrometry (GC-MS/MS) (Fig. 7b, c and Supplementary Fig. 14). Supplementation of 30-nm Q10 clearly increased the concentrations of Q10 in the cerebellum (98.36 ± 2.78% (Con); 59.53 ± 2.72% (HU); 147.89 ± 3.37% (Q10); *p* < 0.001, unpaired *t*-test) (Fig. 7d). FDH activity in the muscle (99.211 ± 1.67% (Con); 77.51 ± 2.71% (HU); 90.11 ± 2.57% (RL); *p* < 0.001, unpaired *t*-test) and cerebellum (98.36 ± 2.78% (Con); 63.46 ± 1.71% (HU); 78.58 ± 2.28% (RL); *p* < 0.001, unpaired *t*-test) in the RL-treated HU mice was markedly elevated compared to the HU mice (Fig. 7e). A previous study indicated that the cysteine 97 and 100 residues were the sites of FDH activated by 630-nm red light^43^ (Fig. 7f and Supplementary Fig. 15).

By using Fluo-HPLC to quantify the concentrations of formaldehyde in the muscle and cerebellum, we found that the three methods of scavenging formaldehyde could markedly reduce the levels of cerebellar formaldehyde (0.4016 ± 0.0059 mM (Con); 1.365 ± 0.0253 mM (HU); 0.8658 ± 0.0569 mM (Q10); 0.8001 ± 0.0558 mM (RL); 0.7276 ± 0.0613 mM (Q10 + RL); *p* < 0.001; one-way ANOVA) (Fig. 7g). In particular, the combination of 30-nm Q10 and RL had a better effect in enhancing formaldehyde metabolism in the muscle (0.2141 ± 0.0274 mM (Con); 0.3445 ± 0.0154 mM (HU vs. Con; *p* = 0.0020); 0.2616 ± 0.0154 mM (Q10 vs. Con; *p* = 0.0182); 0.3051 ± 0.0221 mM (RL vs. Con; *p* = 0.1757); 0.2074 ± 0.0124 mM (Q10 + RL vs. Con; *p* < 0.0001); one-way ANOVA) (Fig. 7h). Consistently, the HU mice treated with Q10 and RL had better abilities to maintain balance and coordinate motor functions than those treated with Q10 or RL alone (263.51 ± 8.72 s (Con); 169.75 ± 15.06 s (HU); 231.75 ± 5.89 s (Q10); 228.62 ± 8.25 s (RL); 263.01 ± 6.48 s (Q10 + RL); *p* < 0.01, *p* < 0.001; one-way ANOVA) (Fig. 7i). The three methods had similar effects in improving sensorimotor functions (14.15 ± 0.51 s (Con); 21.63 ± 1.32 s (HU); 16.24 ± 1.16 s (Q10); 14.63 ± 0.65 s (RL); 16.12 ± 0.52 s (Q10 + RL); *p* < 0.001; one-way ANOVA) (Fig. 7j). Meanwhile, we also found that these three methods could rescue muscle atrophy (Supplementary Fig. 16a), and restore muscular Q10 levels (96.68 ± 2.53% (Con); 68.68 ± 3.57% (HU); 158.79 ± 5.29% (Q10); *p* < 0.001, unpaired *t-*test) (Supplementary Fig. 16b). In addition, these three kinds of treatments also inhibited HU-induced Ca^2+^ influx in the muscle and brains, decreased the expression and activity of SSAO, increased the activity of FDH, and reduced the vacuolization of neurons in the cerebellum (Supplementary Figs. 17–18). These data indicated that the combination of Q10 and RL effectively alleviated motor deficits.

## Discussion

In the present study, we found that HU stress could activate the formaldehyde-generating enzymes, including SSAO and SARDH, directly cause formaldehyde accumulation in the gastrocnemius muscle and the FN of the cerebellum. In addition, hydrogen peroxide (H_2_O_2_), a product of SSAO, could inhibit the activity of the specific formaldehyde-degrading enzyme- FDH^40^. Subsequently, muscular formaldehyde overload directly caused gait instability; especially, cerebellar formaldehyde accumulation induced neuron death and acute cerebellar ataxia. However, scavenging formaldehyde in the muscle and cerebellum could rescue motor deficits (Supplementary Fig. 19).

Muscle atrophy and osteoporosis induced by the weightlessness of space are widely considered to be the main cause for motor deficits in astronauts^44,45^. Recent studies have found that excessive formaldehyde directly leads to muscle atrophy and osteoporosis in the mice with double-knockout of ADH5 and ALDH2^17,18^. The results of in vitro experiments showed that formaldehyde exposure not only inhibits the proliferation of muscular cells and osteoblast but also induces the death of these dells^46,47^. Remarkably, excessive formaldehyde leads to a decline in the total body weight and organ weights^48^, and amyotrophic lateral sclerosis including muscle atrophy^49,50^. Formaldehyde can inhibit potassium currents of the skeletal muscle^51,52^. In this study, formaldehyde directly caused muscle atrophy associated with gait instability. Disquietingly, although microgravity-related muscle atrophy could be gradually recovered by 2-week sport training, motor deficits still existed in model rats exposed to microgravity^9,10^. One reason may be due to that the expression of FDH in the brain is markedly lower than that in the peripheral organs^33^. Thus, the impairment of central cerebellum would be more difficult to reverse than that of the peripheral amyotrophic muscles.

Substantial evidence has shown that the accumulated formaldehyde directly damages brain functions^53^. Clinical studies have found that mutations in the ataxia telangiectasia mutated (*ATM*) gene in patients contribute to sporadic or familial idiopathic ataxia^54^. ATM proteins are expressed in the fastigial nucleus (FN) of the cerebellum^55^. An *ATM* gene mutation in mice was found to cause symptoms mimicking the childhood neurodegenerative disease ataxia-telangiectasia^56^. Hence, ATM in the FN plays a crucial role in the pathological process of cerebellar ataxia^57^. Unsurprisingly, excessive formaldehyde had been found to markedly inactivate ATM protein and induce cell death^58^. Exogenous formaldehyde and methanol directly causes cerebellar ataxia in mice^15^ and humans^16^. Formaldehyde can cause a reduction in the numbers and sizes of Purkinje cells and granular cells, and in the thickness of the granular layer in mice^59^. Thus, formaldehyde-inactivated ATM is a direct factor for the death of the cerebellar neurons and the occurrence of acute ataxia.

In summary, HU stress leads to motor disorders by inducing formaldehyde accumulation in the muscle and cerebellum. Although there is no suitable method to detect the reaction products of formaldehyde with Q10, supplementation of the formaldehyde scavenger- Q10, or activating FDH by illumination with 630-nm red light (which can penetrate the 1.2-cm skull and 1.5-cm abdomen which mimicked human samples^43^), reduced formaldehyde levels and restored endogenous Q10 contents in the muscle and cerebellum; subsequently, it alleviated motor deficits (Supplementary Fig. 19). Hence, the reduction of systemic formaldehyde may be a promising strategy for improving astronaut health.

## Methods

### Chemical regents

Benzylamine (50 μM, ^#^B802559-250ml, Maikelin, Shanghai, China); formaldehyde (0, 0.4, 0.75, 1.5 mM, ^#^F1635-25 Ml, Sigma-aldrich, USA); methoxyacetic acid (0.26 mM, ^#^M830809-25g, Maikelin, Shanghai, China); sarcosine (40 mM, ^#^131776-100 G, Sigma-aldrich, USA); semicarbazide (1 mM, ^#^363634-25 G,.Sigma-aldrich, USA); and verapamil (2 μM, ^#^V8140-5g, Solabel, USA).

### Animals

All protocols involving the use of animals were conducted in accordance with the Biological Research Ethics Committee, Capital Medical University, China (AEEI-2016-147). C57BL/6 mice (25 ± 5 g) were obtained from the Experimental Animal Center of Capital Medical University, China; and FDH^−/−^ male adult mice were provided at 2 months of age by the Institute of Zoology, Chinese Academy of Medical Sciences, China. All animals were maintained in cages at room temperature (25 °C) under an alternating 12-h light/dark cycle (lights on at 7:00), with ad libitum access to food and water.

### Generation of FDH^−/−^ mice using the CRISPR/Cas9 technique

1. Flowchart of FDH knockout mice: CRISPR/Cas9 techniques were used to prepare formaldehyde dehydrogenase (FDH) knockout mice. The procedure included: knockout gene information determination, construction linearization of gRNA, gRNA/Cas9 in vitro transcription, prokaryotic fiber injection, birth, and genotype identification of F0 generation mice as the original founders (Supplementary Fig. 3).
2. Gene information: FDH, also named ADH3 alcohol dehydrogenase (class III), chi polypeptide [Mus musculus (house mouse)] *Gene ID: 11532*, Official Full Name: alcohol dehydrogenase-5 (class III).
3. Targeting site selection: we designed four gRNA targets, two located upstream of Exon 5 (E5, 220 bp) and two downstream of Exon 6 (E6, 261 bp), to achieve knockdown of the gene by simultaneous knockdown of E5 and E6. The primer sequences used to construct the gRNA plasmid are shown in Supplementary Figs. 4 and 5. The insertion sites into plasmid pUC57kan-T7-gRNA are described in Supplementary Fig. 6, and then the constructed plasmid Pst1374-N-NLS-flag-linker-Cas9 is shown in Supplementary Fig. 7.
4. Targeting site selection:
  Four gRNA targets were designed, including two located upstream of exon 5 (E5, 220 bp) and two downstream of exon 6 (E6, 261 bp), to knockout FDH gene by simultaneous deleting E5 and E6.
  The primer sequences were used to construct gRNA as following:
  **A**:
  GG GCTCTGATGTGCTTTCTG GGG
  M-Aldh3(5)-E5A-gR-top: TAGGGCTCTGATGTGCTTTCTG
  M-Aldh3(5)-E5A -gR-dow: AAACCAGAAAGCACATCAGAGC
  **B**:
  GG TCCCTGTGCTACTGTGTT AGG
  M-Aldh3(5)-E5B-gR-top: TAGGTCCCTGTGCTACTGTGTT
  M-Aldh3(5)-E5B-gR-dow: AAACAACACAGTAGCACAGGGA
  **C**:
  GG TTCTGAAACAGCTTTGGT AGG
  M-Aldh3(5)-E5C-gR-top: TAGGTTCTGAAACAGCTTTGGT
  M-Aldh3(5)-E5C-gR-dow: AAACACCAAAGCTGTTTCAGAA
  **D**:
  GG GGAATTACAGAAGGGCTC AGG
  M-Aldh3(5)-E5D-gR-top: TAGGGGAATTACAGAAGGGCTC
  M-Aldh3(5)-E5D-gR-dow: AAACGAGCCCTTCTGTAATTCC
5. Microinjection and genotype identification: after gRNA and Cas9 in vitro transcription and microinjection were completed, the first mouse to be born was used for the next genotype identification process. The primer information for genotype identification was as follows: M-FDH (5)-F1: GCAGCCCAGTGAATGTTTGC 64.0; M-FDH (5)-R1: ATACACACTCCATCCTTCGCTCAG 63.9; size: 1.433 kb. By genotype identification, we successfully obtained a knockout mouse. The results of genotype identification are shown in Supplementary Fig. 8. The lower bands of FDH^−/−^ compared to the wild-type band can be clearly seen.
6. Sequence analysis of gene deletion: by TA cloning and sequencing of the missing fragments, the missing sequence of the *FDH* gene is shown below (marked with underlining). *Del 1115BP***:** gataggtccttctctcagagattatactcctacagcaatgtatagaactgtcttctcatttgtagatttattcatgtgtatattttagagtattcatctgagtgtaaacttggatgttgtgtttattagaaaggatggacgtcctgagaagacggctcagttgggaaaattcagattccccagaaagcacatcagagcccacagtagaggcacaagcgtgtgatcccagcacactagggaaatgggaagagaagacacaacgtccagagctcagctcgcccagcctaacacagtagcacagggaccctctcaaacagtgaagcggcagtgaaaacaatcttctgttgggtgtcaccacagcacaaggaagtgtatgaaagggtctcagcattaggacggttgagaaccactgctttggaagttttttgttttagaatacttttctgagtatttgctggtcgttatttttaaaactgatacagagttagggaaatgagcctgcccttccattttccttttcctctgtcttctctagggtcactcaggggaagggattaatgccagatgggactagcagatttacctgcaaaggaaagtctgtttttcacttcatggggactagcacattttccgagtacacagttgtggctgacatctctgttgctaaaatcgatccttcggcccctttggataaagtctgccttctcggctgtggtatttcaactggctacggggctgctgtgaacactgccaaggtaagagcctggccgcggcttccgcttctgtcctcattcagttcagtgagacattgctggcacaccatggccagcctgtgaggctgtgactcttagcgggcagctgacactgtctttgatgataggtggagcctggttctacttgtgccgtctttggcctgggaggagttggactggcagtgatcatgggctgtaaggtggctggtgcatcccggatcattggtatcgacatcaataaagataaattcgcaaaggccaaagaatttggagcctctgaatgtattagcccccaagacttcagtaaatccatccaggaagtcctcgttgagatgacagatgggggcgtggattactcctttgagtgcattggcaacgtgaaggtcatggtgagtacatgtgcccgcattctttttgggctttggtttcctcatgattaaagctttggttctgaaacagctttggtaggaattacttgttgagaaagcaggcgatggcaatagcgccttgaactacttggacctatcgaatggggaattacagaagggctcagggagagatgctcaccaaaatgaaaggcttagtgtgaagggcatgggctttggaactggacacacacctgagcgaaggatggagtgtgtatctagtagtgtgtcaccaacattacatagagtcctgcagtgacttctgacagcttcttgcacggaaatccttaattaggaaaaaagaaacttttagatttatttat
7. Generation and reproduction: after the first mouse was successfully obtained, it was mated with wild-type mice to reproduce the F1 generation of mice for subsequent amplification.

### Model mice of hindlimb unloading

Two-month-old male C57BL mice, weighing 21–22 g, were randomly assigned to the control (Con) and groups of hindlimb unloading (HU) to partially simulate microgravity. To induce muscle unloading, the animals in the HU group were suspended individually in special cages for 14 consecutive days using a method similar to that used previously for mice^19,60^. Briefly, one end of a thin string was attached to the tail by sticking plaster and the other end was attached to the top of the cage. The length of the string was adjusted to allow the animals to move freely on the forelimbs, while the body was inclined at 30–40° from the horizontal plane. All mice had water ad libitum and received 8 g per day of standard rodent chow. The food remaining on the following day was weighed to determine daily food consumption. At the end of the suspension period, the string was unfastened from the mice, which were then deeply anesthetized by an intraperitoneal injection of urethane (1.2 g/kg body weight). Brain samples were used immediately for formaldehyde fluorescence imaging, formaldehyde quantification, or frozen in liquid nitrogen and stored at −80 °C for other studies.

### HU model mice illuminated with 630-nm red light

After the HU model mice were allowed freedom of movement in a box and illuminated for 30 min twice daily using an abdominal light source for 14 consecutive days;^40^ optical power density: 0.5 mW/cm^2^ (Supplementary Fig. 13a). To detect light penetration in the skull of the mice, a spectrophotometric detector (FLA5000+, Hangzhou Crystal Fly Technology Co., Ltd., China) was placed under the mandibula, and a head light source was placed on the skull (Supplementary Fig. 13b). To detect light penetration in the muscle of the mice, a spectrophotometric detector was placed on the back of the muscle, and the light source was placed under the muscle (Supplementary Fig. 13c).

### HU model mice with intragastric administration of 30-nm Q10

After these HU mice were intragastrically administered 30-nm Q10 (200 mg/kg, once daily) for 14 consecutive days. Nano-coenzyme Q10 (Nano-Q10, diameter: 30–40 nm, which can enhance the water solubility and liposolubility of the non-water-soluble Q10) was donated by Beijing Delivery Pharm Technology Co., Ltd., Beijing, China. Nano-Q10 was dissolved at 37 °C in distilled water and immediately given by oral gavage. In the control group, mice were given distilled water at 37 °C by gavage.

### Q10 levels quantified by the method of ECA-fluorescence

The concentrations of Q10 in the muscle and cerebellum of each mouse were quantified by fluorescence spectrophotometry using the ethyl cyanoacetate (FS-ECA) method as described previously^61^. In brief, this method for detecting CoQ10 was based on the principle that the chemical derivative obtained from the interaction between CoQ10 and ethyl cyanoacetate (ECA) could be detected by a fluorescence spectrophotometer (FS-ECA) at *λ*_ex/em_ = 450/515 nm. Because one methoxy group of CoQ10 can be replaced by a moiety of ECA. To obtain calibration curves, CoQ10 standards were prepared with concentrations ranging from 0.00, 0.055, 0.109, 0.218, 0.437 to 1.750 mg/L. To prepare the detected samples, 60 μL standard CoQ10 or clinical samples were added into the solutions containing 60 μL ethanol and 120 μL hexane. Then, these mixed solutions were vortexed for 1 min and centrifuged at 3000 rpm for 2 min. The supernatants were transferred into 120 μL hexane solution and extracted again. The twice extracted supernatants were combined and dried using nitrogen. These precipitants were resolved by 60 μL ethanol, and 20 μL of this solution was added into 220 μL ECA-sensitive reaction system (40 μL ECA + 40 μL 0.5% KOH + 140 μL ethanol) and the mixture was incubated in dark for 30 min. Then the fluoresent intensitiesof the derivatives were quantified by using FS-ECA at *λ*_ex/em_ = 450/515 nm (Multi-Mode Microplate Reader, SpectraMax i3, Molecular Devices, California, USA).

### FDH activity detected by FDH kits

Formaldehyde dehydrogenase (FDH, also named ADH3) activity was measured using a commercially available kit according to the manufacturer’s instructions (^#^K787-100, Biovision, Milpitas, CA).

### SSAO activity quantified by SSAO kits

The semicarbazide-sensitive amine oxidase (SSAO) was detected by using a commercially available kit according to the manufacturer’s instructions (^#^GMS50538.1, Jimei Gene Medicine Technology Co. Ltd, Shanghai, China).

### Injection of formaldehyde into the gastrocnemius muscle

Formaldehyde at 0.4, 0.75, and 1.5 mM (20 μL) or phosphate buffer saline (PBS, as a vehicle control) was slowly injected into the muscle of hindlimb of wild-type mice at a rate of 0.2 μL/minute, respectively. After an additional 5 m (min), to assure adequate diffusion, the needle was slowly retracted. Intramuscular injection was carried out for 14 consecutive days and then for behavior assessments.

### Microinjection of formaldehyde into the bilateral fastigial nucleus

Implant surgery was carried out as described previously^62,63^. Briefly, the left and right fastigial nuclei were implanted (posterior 2.7 mm from lambda, lateral 0.8 mm, and depth 2.3 mm). Implants were secured to the skull using surgical screws and dental cement. A 2-μL volume of formaldehyde (1.5 mM) or phosphate buffer saline (PBS, as a vehicle control) was slowly infused at a rate of 0.2 μL/min. After an additional 5 min, to assure adequate diffusion, the needle was slowly retracted. These infusions were carried out for 7 consecutive days. Animals were allowed to recover for a minimum of 3 days prior to behavior assessments. The traces of formaldehyde injected into fastigial nucleus (FN) were identified by using red fluorescent probe-Dil (^#^C1036, Byotime, China) to mark cell membrane and blue DAPI (^#^C1002, Byotime, China) to stain nucleus.

### Gait analysis in healthy wild-type mice

Gait was analyzed using the DigiGait^TM^ imaging system (^#^Digigait, Mouse Specifics, Boston, USA). Each mouse had to run three times. Every successful run (sequences of at least four consecutive steps) was taken into account for data analysis. The base of support was determined as the distance between the central pads of the hind paws. Stride length was determined as the distance between the central pads of two consecutive footprints at the same side.

### Accelerating rotarod test of mice

The rotarod is used to assess the balance and motor coordination of animals^60,64^. Before any treatment, all animals were given three trials per day with a 5–10 min intertrial rest interval for 3 days to allow them to adapt to the task. The speed of the rotarod accelerated from 4 to 40 rpm over a 5-min period. On the first day, the mice were first placed on the apparatus for 5 min with a constant low speed of rotation (4 rpm). Those that fell from the rod at 4 rpm were placed on it again until they were able to stay for 1 min. The occurrence of two consecutive passive rotations, i.e., without walking but accompanying the rod, was considered as a fall. Each mouse was tested three times per day at days 0 and 14. The duration of time the mouse was able to walk on the rod before falling was recorded (maximum value: 300 s).

### Beam walking test of mice

This method for measuring the sensorimotor function of rodents was described previously^65^. Briefly, the beam was 80 cm long, 8 mm in diameter, and above the ground with a goal box as an escape place. The mice were moved immediately to the beam test after they finished the rotarod test. Two trials were performed per day for each mouse on each of the slender (8 mm) beams. Mice that fell were returned to the position they fell from, with a maximum time of 60 s allowed on the beam. The time mice took to traverse the beam to the end was recorded.

### Brain formaldehyde imaging

To determine whether simulated microgravity increases brain formaldehyde levels, we used a two-photon fluorescent FA probe (Na-FA; 5 μM; *λ*_ex/em_ = 430/550 nm) to detect formaldehyde^21^. Briefly, the brains of mice were rapidly taken out after they were injected with the NaFA probe (10 µM, 0.5 mL) 30 min. Then, each brain sample underwent animal imaging by an in-vivo small animal imaging system (^#^Fx Pro, Carestream Health, USA).

### Cerebellar formaldehyde quantified by Fluo-HPLC

Formaldehyde concentrations were determined by high-performance liquid chromatography with fluorescence detection (Fluo-HPLC)^23,24,40^.

### Immunofluorescence analysis of cerebellar SSAO

After microwave-based antigen retrieval, paraffin sections were incubated first in 10% normal goat serum (Thermo Fisher Scientific) for 30 min and then with a primary rabbit polyclonal antibody against VAP-1/SSAO (1 μg/mL, ^#^ab42885, Abcam, Cambridge, MA) at 4 °C overnight prior to exposure to Alexa Fluor 488 goat anti-mouse IgG (green, 1:400, Thermo Fisher Scientific) for 30 min at room temperature. DAPI (blue, a nucleus dye used to mark neuronal cells) was co-incubated to double-label the cells. Normal rabbit IgG (1 μg/mL, R&D Systems, Minneapolis, MN) was used as a negative control instead of primary antibodies. Photomicrographs were taken with a fluorescence microscope (^#^BZ-9000, BIOREVO, Keyence, Japan).

### Ca^2+^ contents in the muscle and brain detected by kits

Thhe levels of Ca^2+^ in the muscle and brain was detected by using a commercially available QuantiChrom calcium assay kit according to the manufacturer’s instructions (^#^DICA-500, BioAssay Systems, USA).

### Cerebellar granule neurons stained by Nissl solutions

The cellular morphology or activity of granule neurons was detected by Nissl staining solutions according to the manufacturer’s instructions (^#^C0117, Beyotime, China).

### SH-SY5Y and HAECs with simulated microgravity in a RCCS

Human neuroblastoma SH-SY5Y cells were cultured in DMEM with 10% FBS and 1% PS. Primary human aortic endothelial cells (HAECs, ^#^Clonetics, Allschwil, Switzerland) were cultured and passaged in EBM-2 medium (^#^EGM-2 bulletkit, Clonetics, Allcell, USA), as described previously^66^. These two cells were respectively seeded at a density of 2 × 10^5^ cells/mL either in normal gravity in a petri dish for control cells or in simulated microgravity in the Rotary Cell Culture System (^#^RCCS; Synthecon, Inc., Houston, TX) at 20 rpm for 8 h (h) as described previously^67,68^. After simulating microgravity for 8 h, SH-SY5Y cells were incubated with a mitochondrial-targeted formaldehyde fluorescent probe (MT-FA)^29^ or Na-FA^21^ for 30 min to probe the formaldehyde in the mitochondria and cytoplasm and incubated with Fura-2 AM (*λ*_ex/em_ = 340/510 nm) for 30 min to detect the level of calcium ions (Ca^2+^) at 37 °C with 5% CO_2_. Fluorescence imaging of the cells was visualized on a Zeiss confocal microscope (^#^LSM880, Zeiss, USA).

### Quantification of cellular Ca^2+^ levels by laser confocal microscopy

#### *S*canning [Ca^2+^]i by laser confocal microscopy

The Ca^2+^ probe Fura-2 AM was used for the [Ca^2+^i] imaging in the cultured cells as described previously^30,69^. In brief, these cells were loaded with 5 μM Fura-2 AM solution (#S1052, 2 mM/50 μL, Biyuntian, Lo, Shanghai, China) and incubated for 30 min at 37 °C. Fura-2 AM was illuminated at 340 and 380 nm, The equation [Ca^2+^i] = (*R*−*R*_min_)/(*R*_max_−*R*)Sf×Kd can be used to convert the Fura-2 AM ratio values to intracellular calcium concentrations. [Ca^2+^i] is the calcium concentration, *R* is the Fura-2 AM 340/380 ratio, *R*_min_ and *R*_max_ are the 340/380 ratios in the absence of calcium or in the presence of a saturating concentration of calcium respectively; and Sf×Kd is the product of the Kd of Fura-2 AM and a scaling value. To measure *R*_min_, *R*_max_ and Sf×Kd it is necessary to perform in vitro calibration. Changes in cytosolic [Ca^2+^i] concentration in these cells were measured with a confocal laser scanning microscope (Leica Company Ltd., Germany).

### Detection of mitochondrial or cytoplasmic FA levels by laser confocal microscopy

Formaldehyde concentrations in the mitochondria or cytoplasm of these cells were determined using an MT-FA or NaFA probe, respectively, as described previously^21,29^. In brief, to quantify cytoplasmic FA concentrations by using 5 μM NaFA probe and detect mitochondrial FA levels by using 5 μM MT-FA probes in these cultured cells. The culture medium of the cells was changed to a fresh media with 5 μM probe, and then incubated for 30 min. Subsequently, the medium was removed and washed three times with PBS to remove the excess probe. Then imaging studies of cytoplasmic FA were conducted by using the excitation at the channel 430 nm and emission collected from 500 to 550 nm. The imaging studies of mitochondrial FA were conducted at *λ*_ex/em_ = 440/550 nm. The changes in FA concentrations in these cells were quantified by using a confocal laser scanning microscope (Leica Company Ltd., Germany).

### Culture of cerebellar granule neurons

Cerebellar granule neurons were prepared from 3-day-old C57BL/6 mice as previously described^57,70^. Briefly, freshly dissected cerebella were dissociated and the cells seeded at a density of 1 × 10^5^ cells/mL on poly-l-lysine-coated dishes in basal medium Eagle supplemented with 10% FBS, 25 mM KCL, and 0.1 mg/mL gentamicin. Cytosine arabinoside (10 mM) was added to the culture medium 24 h after initial plating. All experiments used neurons after 7–8 days in vitro.

### Assessment of neuronal viability using CCK-8 kits

Cell viability of the cerebellar granule neurons was determined using Cell Counting Kit‑8 (CCK‑8) according to the manufacturer’s instructions (^#^E1CK-000208-10, EnoGene, China).

### Reaction between Q10 and formaldehyde identified by GC-MS/MS

Coenzyme Q10 and FA solutions were purchased from Sigma-Aldrich, USA. The standard samples of Q10 (100 μM) and FA (400 μM) were co-incubated in 100% alcohol solution for 72 h at 37 °C. Then the mixed solutions were examined by gas chromatography-tandem mass spectrometry (GC-MS/MS) (^#^MicrOTOF-Q, Bruker, Germany).

### Statistics and reproducibility

The differences between the different groups within each day were analyzed by one-way ANOVA with Bonferroni’s correction. For other experiments, statistical significance was determined by means of the unpaired *t*-test (for independent or dependent samples, as appropriate) with *p* < 0.05 (two-tailed) considered as a significant difference. All data are reported as the mean ± standard errors.

### Reporting summary

Further information on research design is available in the Nature Research Reporting Summary linked to this article.

## Supplementary information

Supplementary Information

Description of additional supplementary files

Supplementary data 1

Supplementary Movies 1–9

Reporting Summary

## Data Availability

The data that support the findings of this study are available from the corresponding author upon reasonable request. Source data of figures and supplementary figures are presented in Supplementary Data 1.

## References

[1] Grigor’ev AI (2006). Aerospace biology and medicine, aerospace biotechnology and astrobiology Russian Federation government program. Aviakosm. Ekolog. Med..

[2] Black FO (1999). Disruption of postural readaptation by inertial stimuli following space flight. J. Vestib. Res..

[3] Fujii MD (1992). Neurology of microgravity and space travel. Neurol. Clin..

[4] Paloski WH (1998). Vestibulospinal adaptation to microgravity. Otolaryngol. Head Neck Surg..

[5] Paloski WH (1992). Recovery of postural equilibrium control following spaceflight. Ann. N. Y. Acad. Sci..

[6] News, I. T. *After spending 197 days in space, NASA astronaut Andrew J. Feustel struggles to walk again on Earth* https://www.indiatvnews.com/science/news-after-spending-197-days-in-space-nasa-astronautandrew-j-feustel-struggles-to-walk-again-on-earth-video-inside-495550 (2018).

[7] Salazar AP (2020). Neural working memory changes during a spaceflight analog with elevated carbon dioxide: a pilot study. Front. Syst. Neurosci..

[8] Holstein GR (1999). Anatomical observations of the rat cerebellar nodulus after 24 h of spaceflight. J. Gravit. Physiol..

[9] Ishihara A (2000). Comparison of the response of motoneurons innervating perineal and hind limb muscles to spaceflight and recovery. Muscle Nerve.

[10] Tajino J (2015). Discordance in recovery between altered locomotion and muscle atrophy induced by simulated microgravity in rats. J. Mot. Behav..

[11] Zhang XY (2016). Cerebellar fastigial nucleus: from anatomic construction to physiological functions. Cerebellum Ataxias.

[12] Schmahmann JD (2007). The neuropsychiatry of the cerebellum - insights from the clinic. Cerebellum.

[13] Zhang Y (2012). Simulated microgravity affects semicarbazide-sensitive amine oxidase expression in recombinant Escherichia coli by HPLC-ESI-QQQ analysis. Appl. Microbiol. Biotechnol..

[14] Mackey VR (2012). Prenatal exposure to methanol as a dopamine system sensitization model in C57BL/6J mice. Life Sci..

[15] Maronpot RR (1986). Toxicity of formaldehyde vapor in B6C3F1 mice exposed for 13 weeks. Toxicology.

[16] Klein KA (2017). Optical coherence tomography findings in methanol toxicity. Int. J. Retina Vitreous.

[17] Tan W (2020). Formaldehyde causes bone marrow failure linked to transcriptional reprogramming or metabolic deficiency. Mol. Cell.

[18] Dingler FA (2020). Two aldehyde clearance systems are essential to prevent lethal formaldehyde accumulation in mice and humans. Mol. Cell.

[19] Tajino J (2015). Intermittent application of hypergravity by centrifugation attenuates disruption of rat gait induced by 2 weeks of simulated microgravity. Behav. Brain Res..

[20] Canu MH (1998). Effect of hindlimb unloading on interlimb coordination during treadmill locomotion in the rat. Eur. J. Appl. Physiol. Occup. Physiol..

[21] Tang Y (2016). Development of a two-photon fluorescent probe for imaging of endogenous formaldehyde in living tissues. Angew. Chem. Int Ed. Engl..

[22] Ai L (2019). A rapid and sensitive fluorescence method for detecting urine formaldehyde in patients with Alzheimer’s disease. Ann. Clin. Biochem..

[23] Luo W (2001). Determination of formaldehyde in blood plasma by high-performance liquid chromatography with fluorescence detection. J. Chromatogr. B Biomed. Sci. Appl..

[24] Tong Z (2017). Urine formaldehyde predicts cognitive impairment in post-stroke dementia and Alzheimer’s disease. J. Alzheimers Dis..

[25] Ai L (2019). Endogenous formaldehyde is a memory-related molecule in mice and humans. Commun. Biol..

[26] Yu PH (1997). Formation of formaldehyde from adrenaline in vivo; a potential risk factor for stress-related angiopathy. Neurochem. Res..

[27] Deng Y (1999). Simultaneous determination of formaldehyde and methylglyoxal in urine: involvement of semicarbazide-sensitive amine oxidase-mediated deamination in diabetic complications. J. Chromatogr. Sci..

[28] Thompson CM (2010). Formaldehyde dehydrogenase: beyond phase I metabolism. Toxicol. Lett..

[29] An XU (2018). Development of a mitochondrial-targeted two-photon fluorescence turn-on probe for formaldehyde and its bio-imaging applications in living cells and tissue. New J. Chem..

[30] Barreto-Chang OdmaraL (2009). Calcium imaging of cortical neurons using Fura-2 AM. J. Vis. Exp..

[31] Fischer MJ (2015). Formalin evokes calcium transients from the endoplasmatic reticulum. PLoS ONE.

[32] Boor PJ (1992). Methylamine metabolism to formaldehyde by vascular semicarbazide-sensitive amine oxidase. Toxicology.

[33] Harris C (2003). Methanol metabolism and embryotoxicity in rat and mouse conceptuses: comparisons of alcohol dehydrogenase (ADH1), formaldehyde dehydrogenase (ADH3), and catalase. Reprod. Toxicol..

[34] Teng S (2001). The formaldehyde metabolic detoxification enzyme systems and molecular cytotoxic mechanism in isolated rat hepatocytes. Chem. Biol. Interact..

[35] Bowerman M (2012). Fasudil improves survival and promotes skeletal muscle development in a mouse model of spinal muscular atrophy. BMC Med..

[36] Wang Z (2020). Intramuscular brown fat activation decreases muscle atrophy and fatty infiltration and improves gait after delayed rotator cuff repair in mice. Am. J. Sports Med..

[37] Gironi M (2004). Late-onset cerebellar ataxia with hypogonadism and muscle coenzyme Q10 deficiency. Neurology.

[38] Folkers K (1995). Two successful double-blind trials with coenzyme Q10(vitamin Q10) on muscular dystrophies and neurogenic atrophies. Biochim. Biophys. Acta.

[39] Franklin, G. P. et al. *The Mouse Brain In Stereotaxic Coordinates* 2nd edn, Vol. 2. p. 1–350 (Academic Press, A Harcourt Science and Technology Company, 2001).

[40] Zhang J (2019). Illumination with 630 nm red light reduces oxidative stress and restores memory by photo-activating catalase and formaldehyde dehydrogenase in SAMP8 mice. Antioxid. Redox Signal..

[41] Momiyama Y (2014). Serum coenzyme Q10 levels as a predictor of dementia in a Japanese general population. Atherosclerosis.

[42] Tong ZQ (2011). Urine formaldehyde level is inversely correlated to mini mental state examination scores in senile dementia. Neurobiol. Aging.

[43] Xiangpei Yue (2019). New insight into Alzheimer’s disease: light recovers Aβ-obstructed interstitial fluid flow and ameliorates memory decline in APP/PS1 mice. Alzheimers Dement..

[44] Ohshima H (2010). [Musculoskeletal rehabilitation and bone. Musculoskeletal response to human space flight and physical countermeasures]. Clin. Calcium.

[45] Chen, Z. et al. Recombinant irisin prevents the reduction of osteoblast differentiation induced by stimulated microgravity through increasing beta-catenin expression. *Int. J. Mol. Sci.* **21**, 1259 (2020). 10.3390/ijms21041259 PMC7072919 32070052

[46] Ho YC (2007). Cytotoxicity of formaldehyde on human osteoblastic cells is related to intracellular glutathione levels. J. Biomed. Mater. Res B Appl. Biomater..

[47] Teng X (2021). Inhibition of osteoblast proliferation and migration by exogenous and endogenous formaldehyde. Hum. Exp. Toxicol..

[48] Nakamura J (2020). The failure of two major formaldehyde catabolism enzymes (ADH5 and ALDH2) leads to partial synthetic lethality in C57BL/6 mice. Genes Environ..

[49] Lee A (2020). Plasma from some patients with amyotrophic lateral sclerosis exhibits elevated formaldehyde levels. J. Neurol. Sci..

[50] Seals RM (2017). Occupational formaldehyde and amyotrophic lateral sclerosis. Eur. J. Epidemiol..

[51] Hutter OF (1969). Potassium conductance of skeletal muscle treated with formaldehyde. Nature.

[52] Hutter OF (1979). A dual effect of formaldehyde on the inwardly rectifying potassium conductance in skeletal muscle. J. Physiol..

[53] Rana I (2021). Formaldehyde and brain disorders: a meta-analysis and bioinformatics approach. Neurotox. Res..

[54] Hassin-Baer S (1999). Absence of mutations in ATM, the gene responsible for ataxia telangiectasia in patients with cerebellar ataxia. J. Neurol..

[55] Oka A (1998). Expression of the ataxia-telangiectasia gene (ATM) product in human cerebellar neurons during development. Neurosci. Lett..

[56] Barlow C (2000). ATM is a cytoplasmic protein in mouse brain required to prevent lysosomal accumulation. Proc. Natl. Acad. Sci. USA.

[57] Dar I (2006). Analysis of the ataxia telangiectasia mutated-mediated DNA damage response in murine cerebellar neurons. J. Neurosci..

[58] Ortega-Atienza S (2016). ATM and KAT5 safeguard replicating chromatin against formaldehyde damage. Nucleic Acids Res..

[59] Mohammadi S (2014). Effect of selenium on neurotoxicity in adult male mice exposed to formaldehyde. Electron. Physician.

[60] Serradj N (2016). Postnatal training of 129/Sv mice confirms the long-term influence of early exercising on the motor properties of mice. Behav. Brain Res..

[61] Fei, X. et al. A rapid and non-invasive fluorescence method for quantifying coenzyme Q10 in blood and urine in clinical analysis. *J. Clin. Lab Anal.* **34**, e23130 (2019). 10.1002/jcla.23130 PMC7171321 31876061

[62] Bohne P (2019). A new projection from the deep cerebellar nuclei to the hippocampus via the ventrolateral and laterodorsal thalamus in mice. Front. Neural Circuits.

[63] Gao Z (2018). A cortico-cerebellar loop for motor planning. Nature.

[64] Piot-Grosjean O (2001). Assessment of sensorimotor and cognitive deficits induced by a moderate traumatic injury in the right parietal cortex of the rat. Neurobiol. Dis..

[65] Stanley JL (2005). The mouse beam walking assay offers improved sensitivity over the mouse rotarod in determining motor coordination deficits induced by benzodiazepines. J. Psychopharmacol..

[66] Qureshi AI (2001). Spontaneous intracerebral hemorrhage. New Engl. J. Med..

[67] Wang J (2009). Simulated microgravity promotes cellular senescence via oxidant stress in rat PC12 cells. Neurochem. Int..

[68] Wang X (2016). Effects of simulated microgravity on human brain nervous tissue. Neurosci. Lett..

[69] Tong ZQ (2010). Tumor tissue-derived formaldehyde and acidic microenvironment synergistically induce bone cancer pain. PLoS ONE.

[70] Du Y (1997). Activation of a caspase 3-related cysteine protease is required for glutamate-mediated apoptosis of cultured cerebellar granule neurons. Proc. Natl. Acad. Sci. USA.

