# Author Correction: Accumulation of formaldehyde causes motor deficits in an in vivo model of hindlimb unloading

**DOI:** 10.1038/s42003-021-02713-x

**Published:** 2021-10-01

**Authors:** Dandan Yao, Qingyuan He, Shangying Bai, Hang Zhao, Jun Yang, Dehua Cui, Yan Yu, Xuechao Fei, Yufei Mei, Ye Cheng, Shi Yan, Nayan Huang, Yalan Di, Xianjie Cai, Rui Wang, Yajuan Gao, Fangrao Cheng, Shengjie Zhao, Xu Yang, Xiang Cai, Hongbin Han, Jihui Lyu, Zhiqian Tong

**Affiliations:** 1 Beijing Institute of Brain Disorders, Laboratory of Brain Disorders, Ministry of Science and Technology, Collaborative Innovation Center for Brain Disorders, Capital Medical University, Beijing, China; 2 Center for Cognitive Disorders, Beijing Geriatric Hospital, Beijing, China; 3 Institute of Aging, Key Laboratory of Alzheimer’s Disease of Zhejiang Province, School of Mental Health, Wenzhou Medical University, Wenzhou, China; 4 Department of Radiology, Peking University Third Hospital, Beijing, Key Laboratory of Magnetic Resonance Imaging Equipment and Technique, Beijing, China; 5 Chinese institute of Rehabilitation Science, China Rehabilitation Research Center, Beijing Key Laboratory of Neural Injury and Rehabilitation, Beijing, China; 6 Section of Environmental Biomedicine, Hubei Key Laboratory of Genetic Regulation and Integrative Biology, College of Life Sciences, Central China Normal University, Wuhan, China

**Keywords:** Movement disorders, Cerebellum

Correction to: *Communications Biology* 10.1038/s42003-021-02448-9, published online 19 August 2021.

In this article the author name Shangying Bai was incorrectly written as Shangyin Bai. The original article has been corrected.

