# Supplementary material for: Accumulation of formaldehyde causes motor deficits in an in vivo model of hindlimb unloading
Source: Commun Biol. 2021 Aug 19;4:933. doi: 10.1038/s42003-021-02448-9 (PMC8376875; doi:10.1038/s42003-021-02448-9)
Supplement: Supplementary file 1 — Supplementary Information [file 42003_2021_2448_MOESM1_ESM.pdf]

Supplementary Figure 1

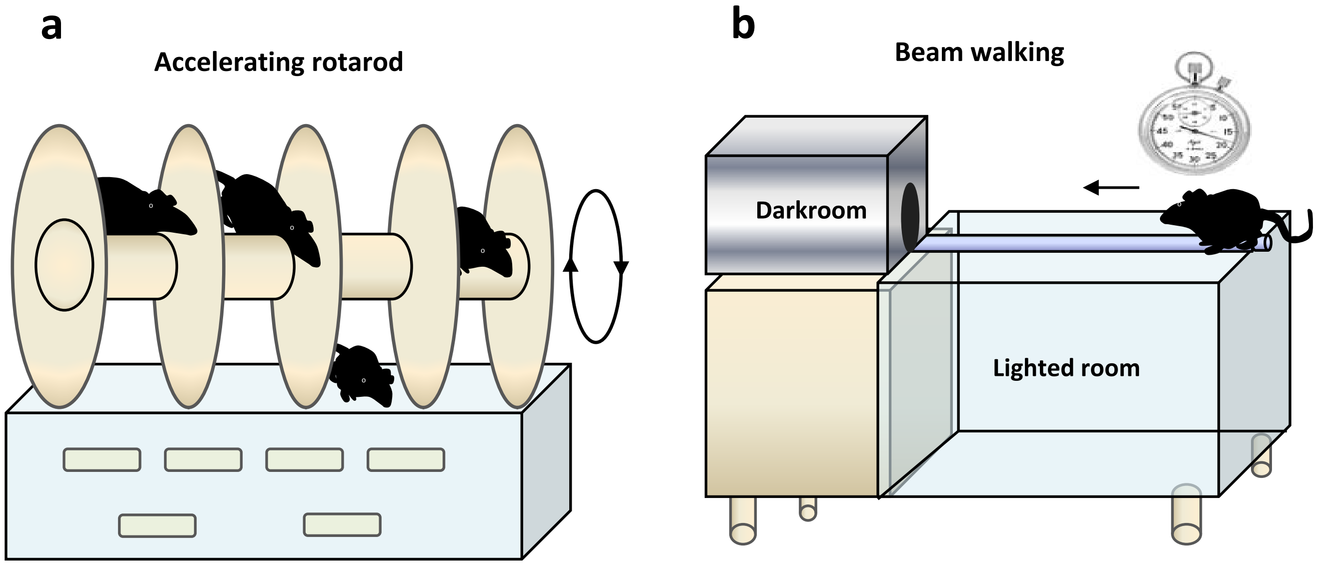

Fig. S1. Motor behaviors assessed by the accelerated rotarod test (a) and bean walking test (b).

Supplementary Figure 2

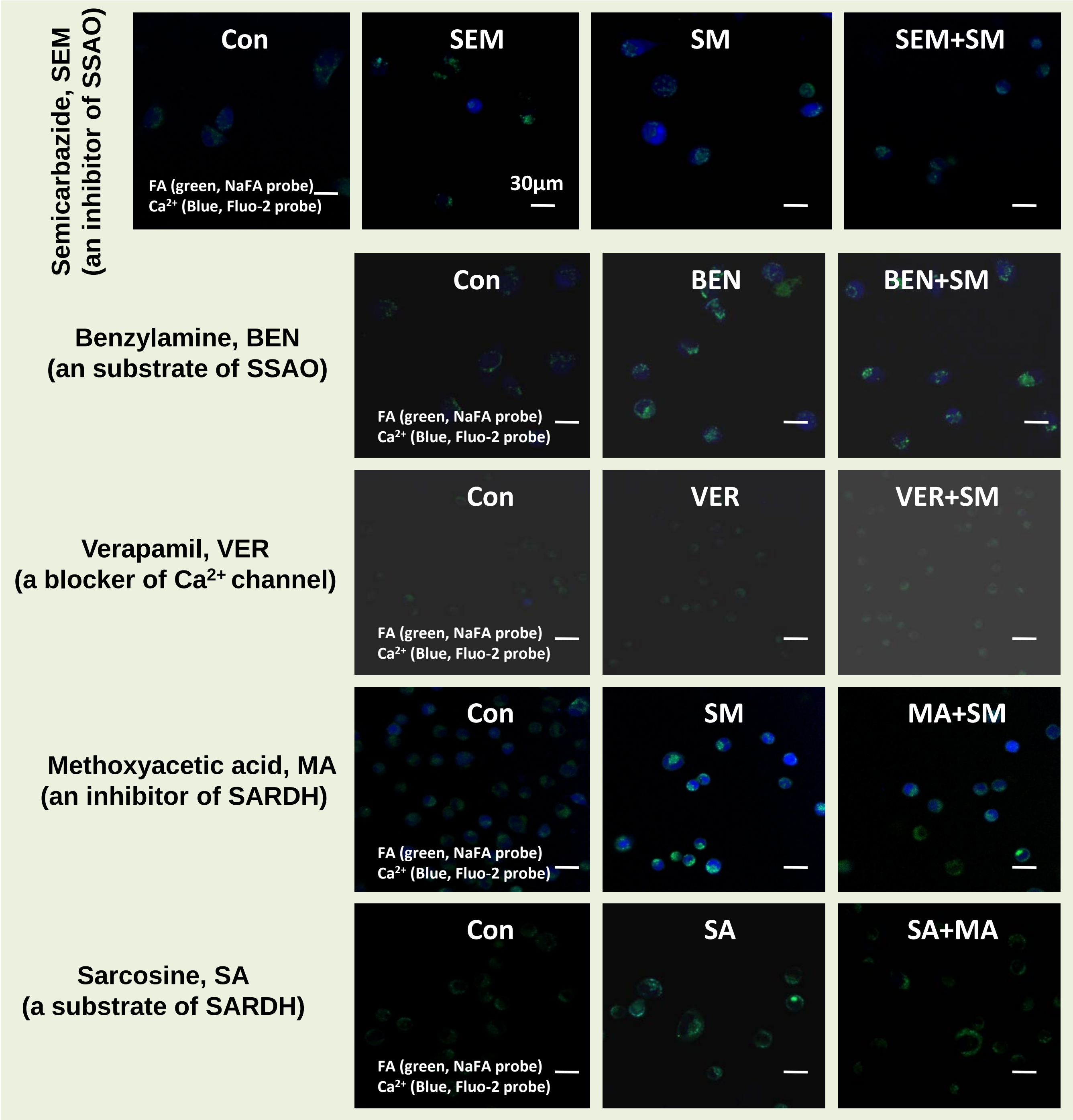

Fig. S2. The effects of the medicines on [Ca^{2+}] influx and intracellular FA generation in the cultured HAECs. BEN: Benzylamine; HAECs: Human aortic endothelial cells; MA: Methoxyacetic acid; SA: Sarcosine; SEM: Semicarbazide; SM: simulated microgravity; VER: Verapamil. Scale bar = 30 μm.

Supplementary Figure 3

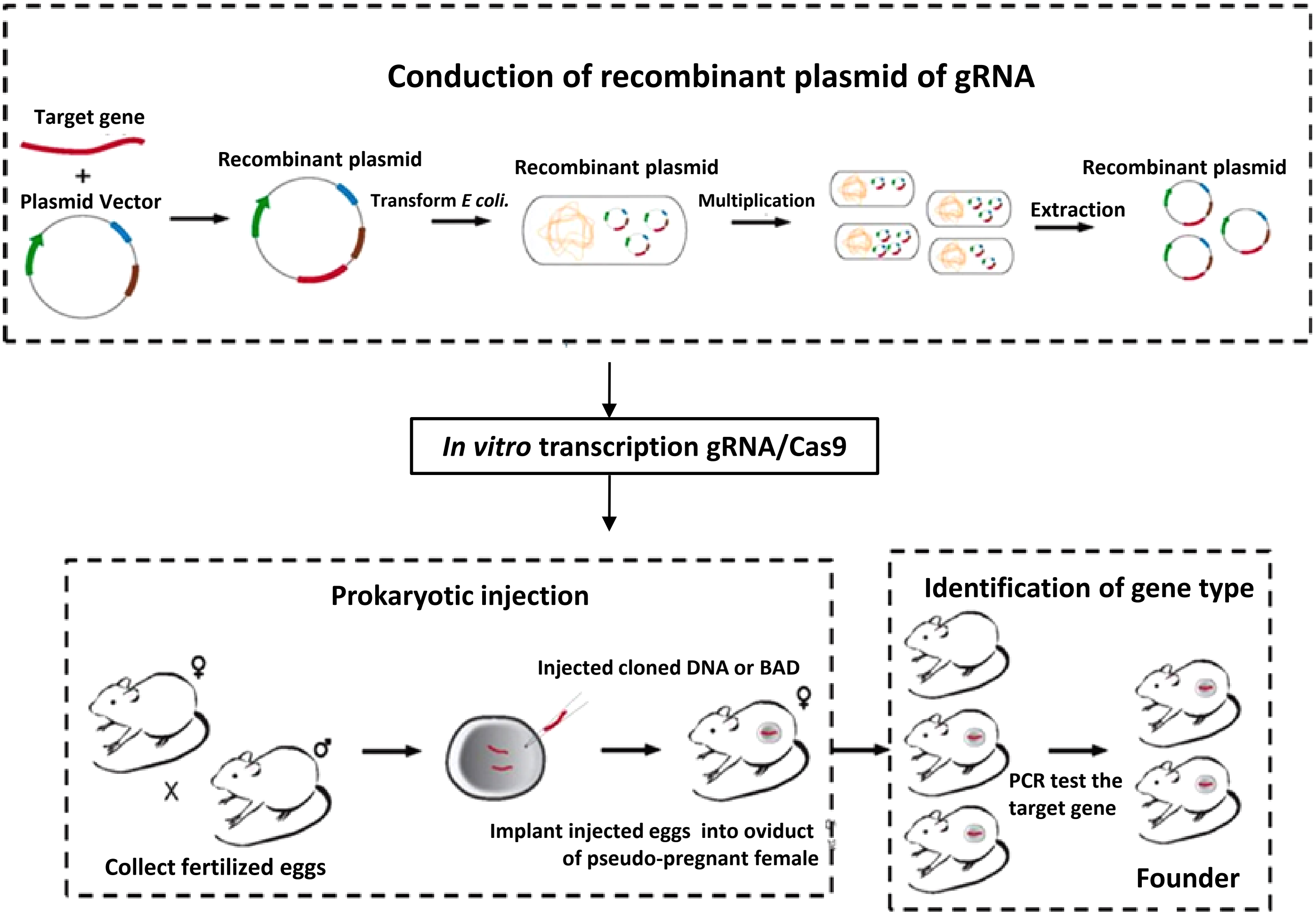

Fig. S3. The technology roadmap of making *FDH*$^{-/-}$ mice by using CRISPR/Cas9 method.

Supplementary Figure 4

Targeting site selection

Four gRNA targets were designed, including two located upstream of Exon 5 (E5, 220 bp) and two downstream of Exon 6 (E6, 261 bp), to knockout *FDH* gene by simultaneous deleting E5 and E6.

The primer sequences were used to construct gRNA as following:
A:
GG GCTCTGATGTGCTTTCTG GGG
M-Aldh3(5)-E5A-gR-top: TAGGGCTCTGATGTGCTTTCTG
M-Aldh3(5)-E5A -gR-dow: AAACCAGAAAGCACATCAGAGC
B:
GG TCCCTGTGCTACTGTGTT AGG
M-Aldh3(5)-E5B-gR-top: TAGGTCCCTGTGCTACTGTGTT
M-Aldh3(5)-E5B-gR-dow: AAACAACACAGTAGCACAGGGA
C:
GG TTCTGAAACAGCTTTGGT AGG
M-Aldh3(5)-E5C-gR-top: TAGGTTCTGAAACAGCTTTGGT
M-Aldh3(5)-E5C-gR-dow: AAACACCAAAGCTGTTTCAGAA
D:
GG GGAATTACAGAAGGGCTC AGG
M-Aldh3(5)-E5D-gR-top: TAGGGGAATTACAGAAGGGCTC
M-Aldh3(5)-E5D-gR-dow: AAACGAGCCCTTCTGTAATTCC

Fig. S4. The primer sequences to construct gRNA plasmid.

Supplementary Figure 5

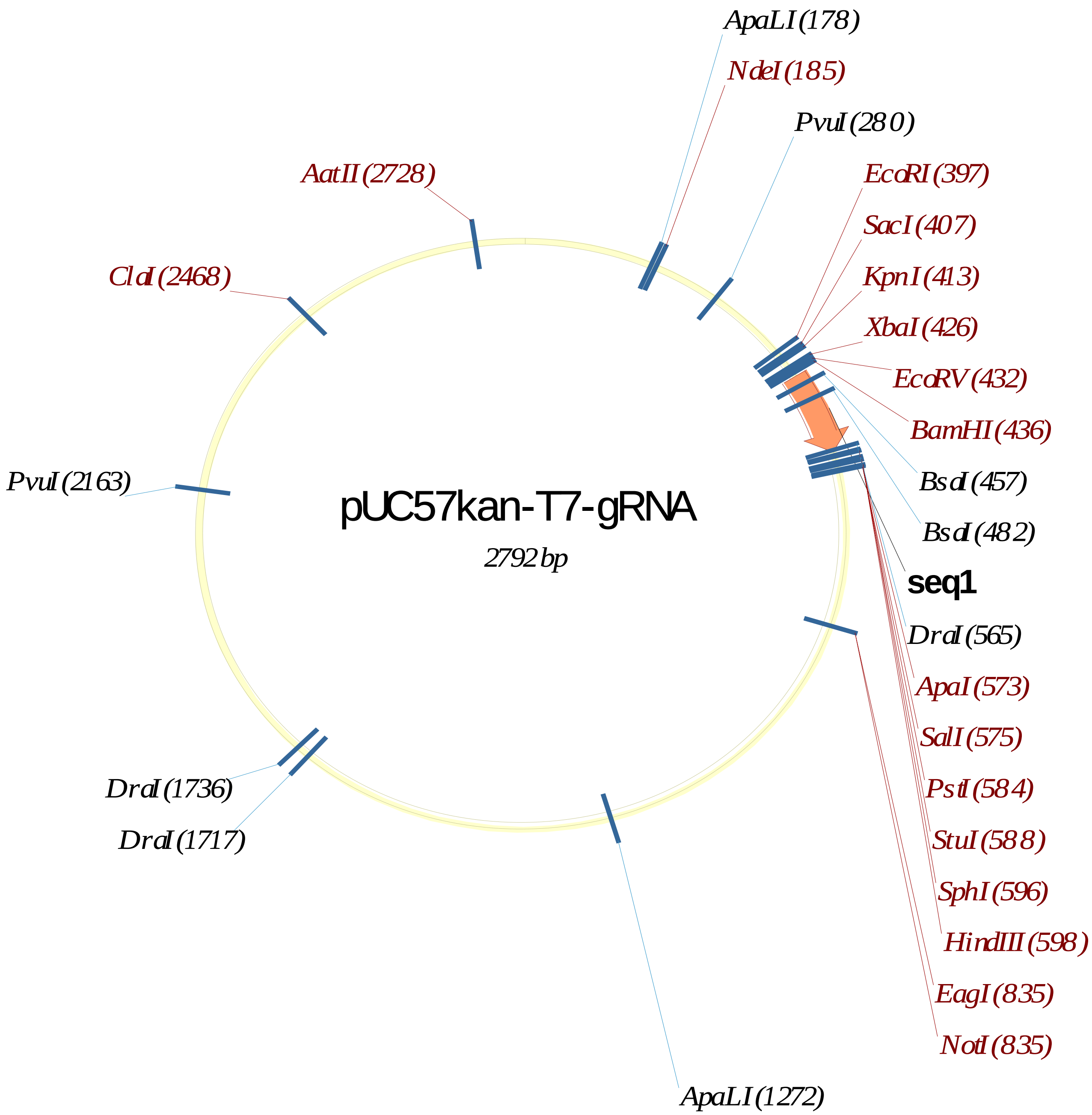

Fig. S5. Construction of plasmid- pUC57kan-T7-gRNA.

Supplementary Figure 6

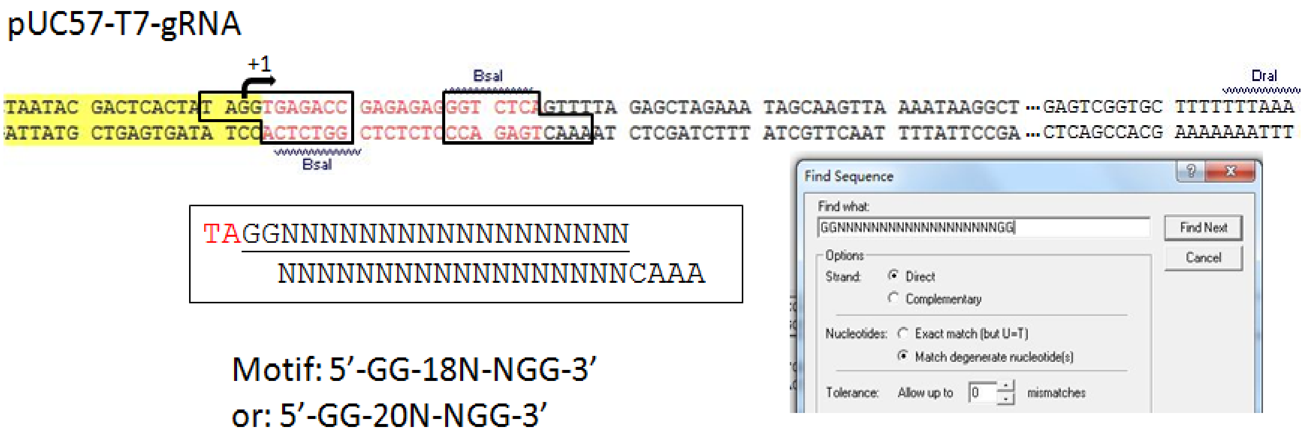

Fig. S6. Mapping of insertion sites into plasmid- pUC57kan-T7-gRNA.

Supplementary Figure 7

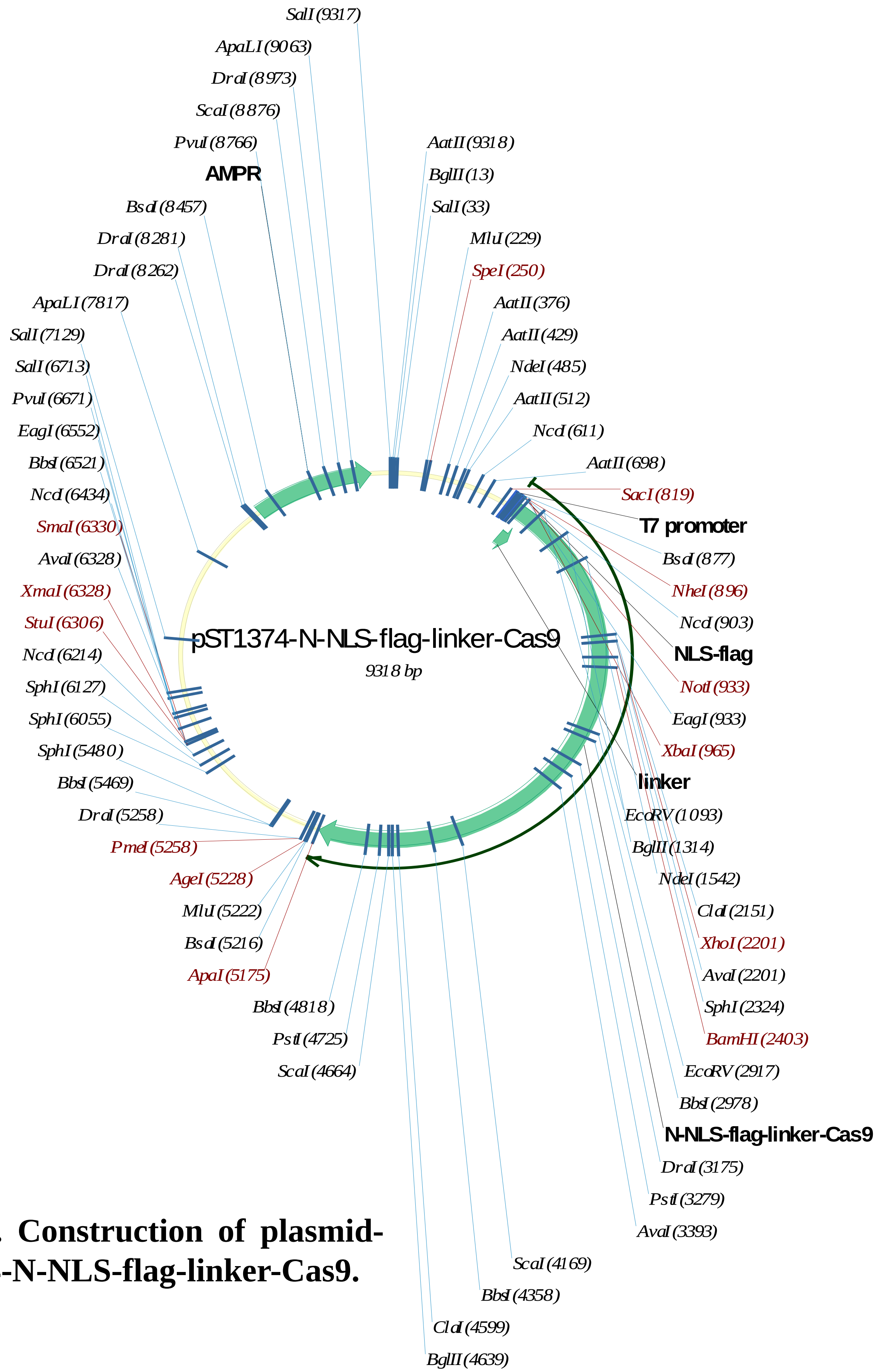

Fig. S7. Construction of plasmid-Pst1374-N-NLS-flag-linker-Cas9.

Supplementary Figure 8

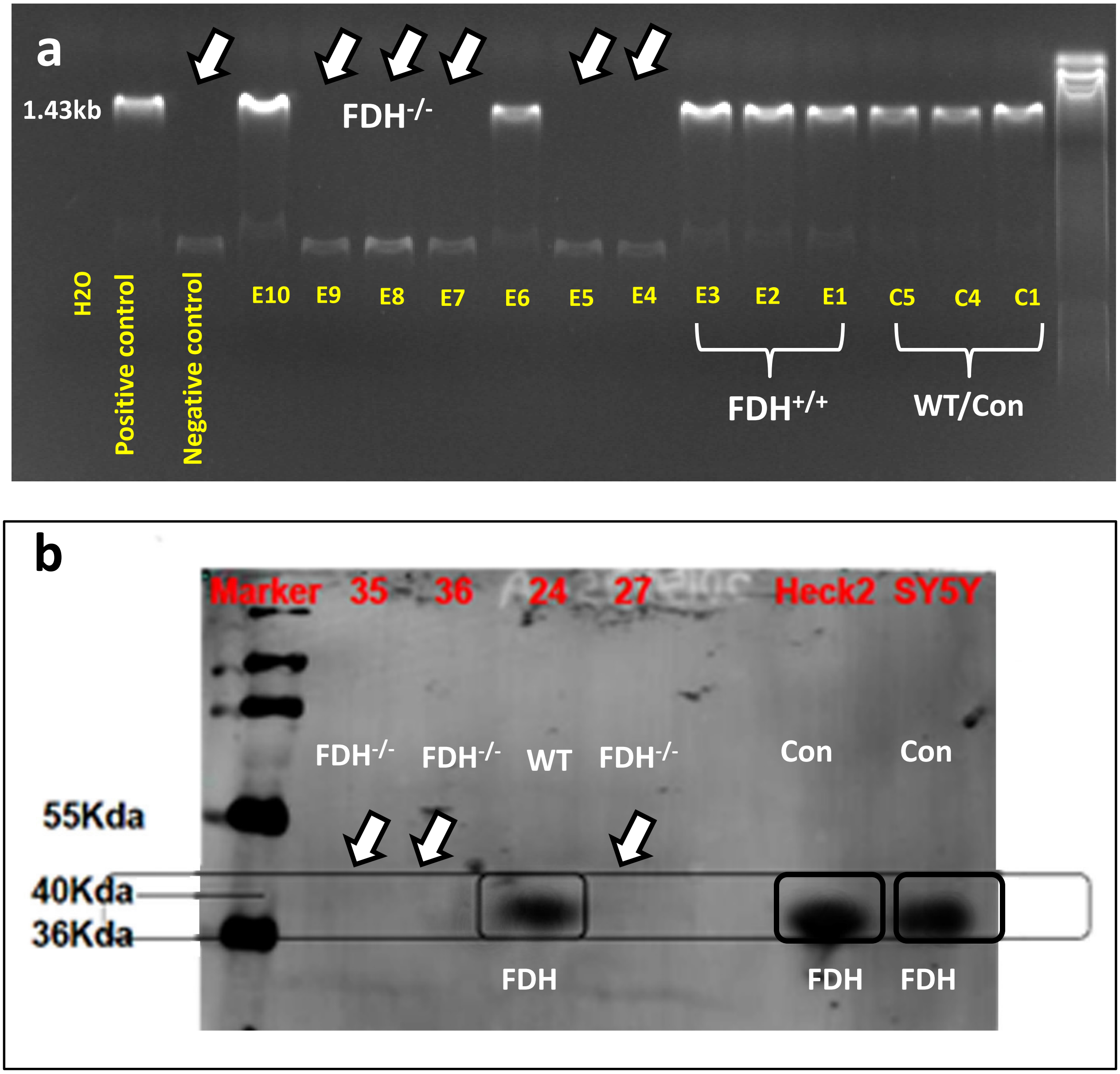

Fig. S8. The DNA with 1.43 kb and protein with 40 Kda of FDH in these *FDH*$^{-/-}$ mice were identified by the methods of reverse transcription- polymerase chain reaction (RT-PCR) (A) and Western blotting (B) (The proteins extracted from 24# wild-type mice, cultured Heck2 and SH-SY5Y cells acted as positive controls).

Supplementary Figure 9

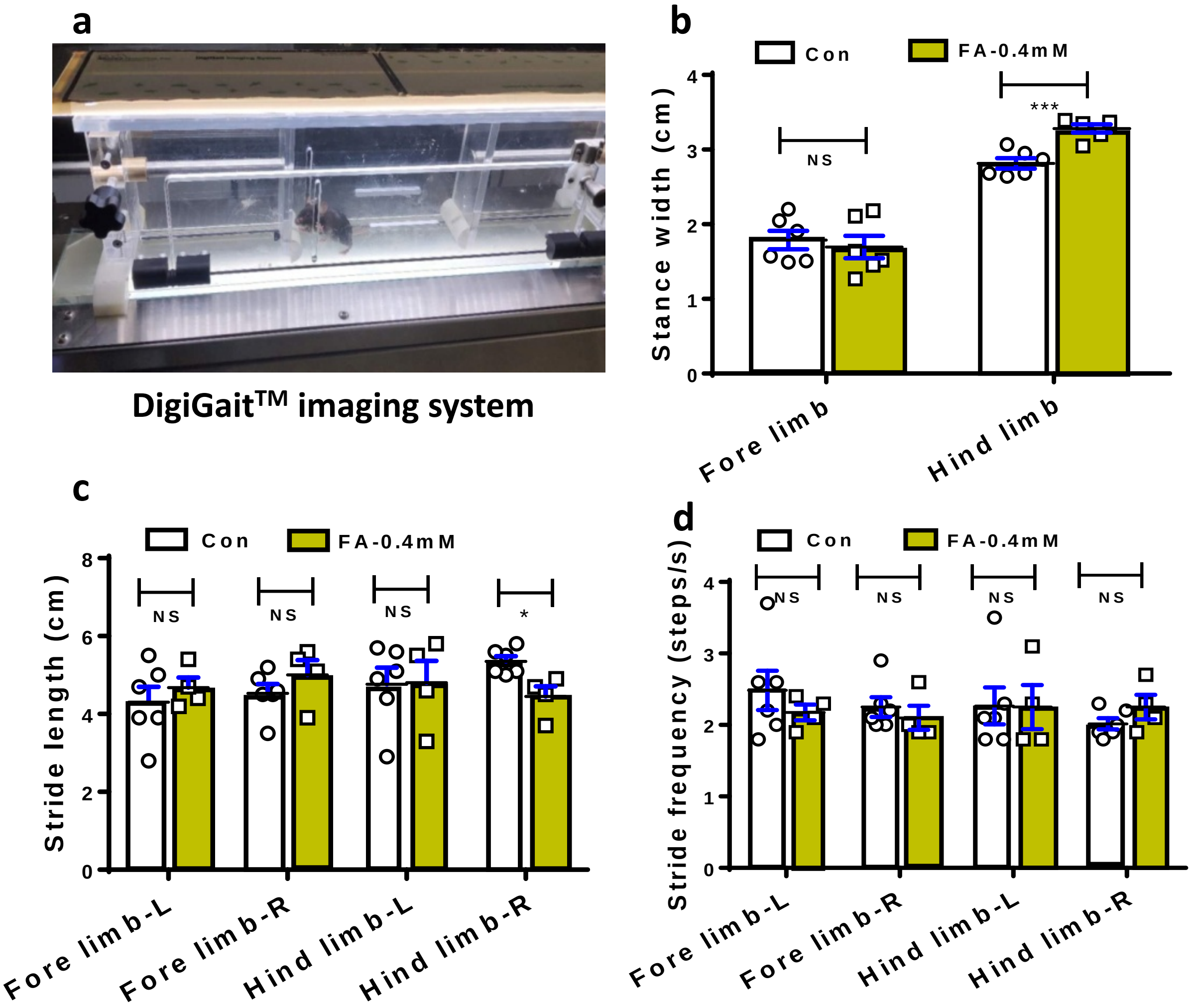

Fig. S9. Gait analysis of control and model mice intramuscularly injected with formaldehyde for 14 consecutive days. (**a**) Gait analysis by the DigiGait™ imaging system. (**b**) Statistical analysis of stance width (n=6). Con: wild-type mice injected with PBS; FA-0.4mM: the healthy adult mice intramuscularly injected with 0.4 mM formaldehyde. (**c**) Statistical analysis of stride length (n=6). (**d**) Statistical analysis of stride frequency (n=6). Error bars show mean ± SEM; NS: no statistical significance; *p<0.05; ***p<0.001.

Supplementary Figure 10

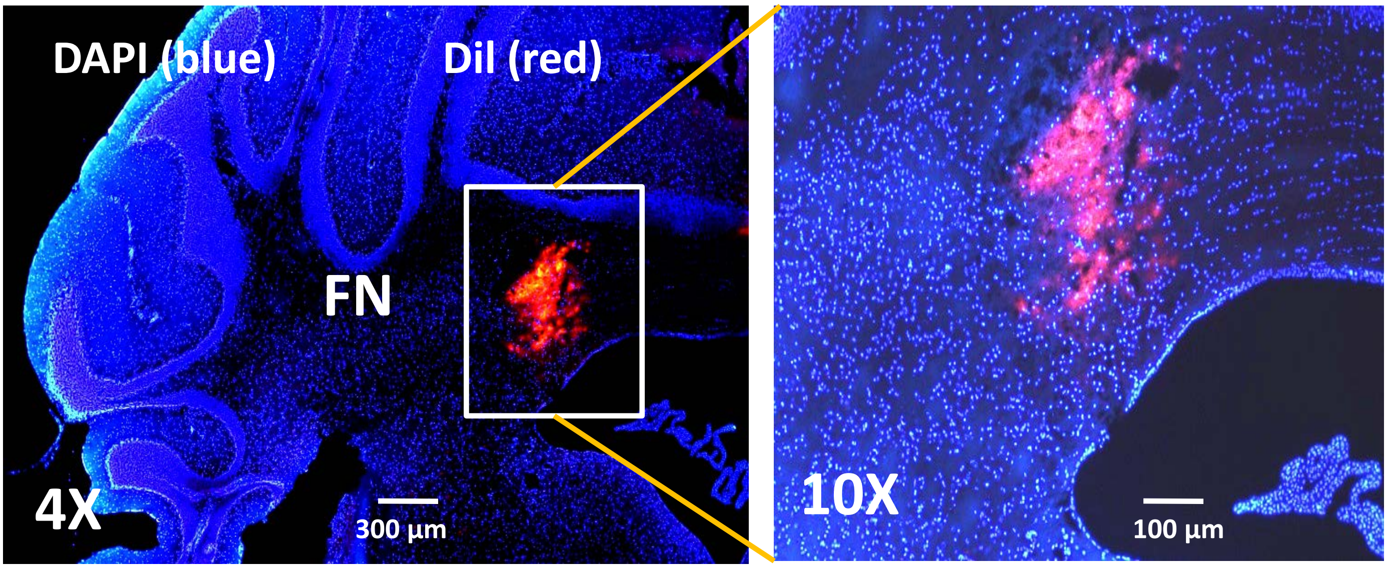

Fig. S10. The injection site of the fastigial nucleus was identified by using red fluorescent probe-Dil to mark cell membrane and blue DAPI to stain nucleus. FN: fastigial nucleus. Scale bar = 300 µm.

Supplementary Figure 11

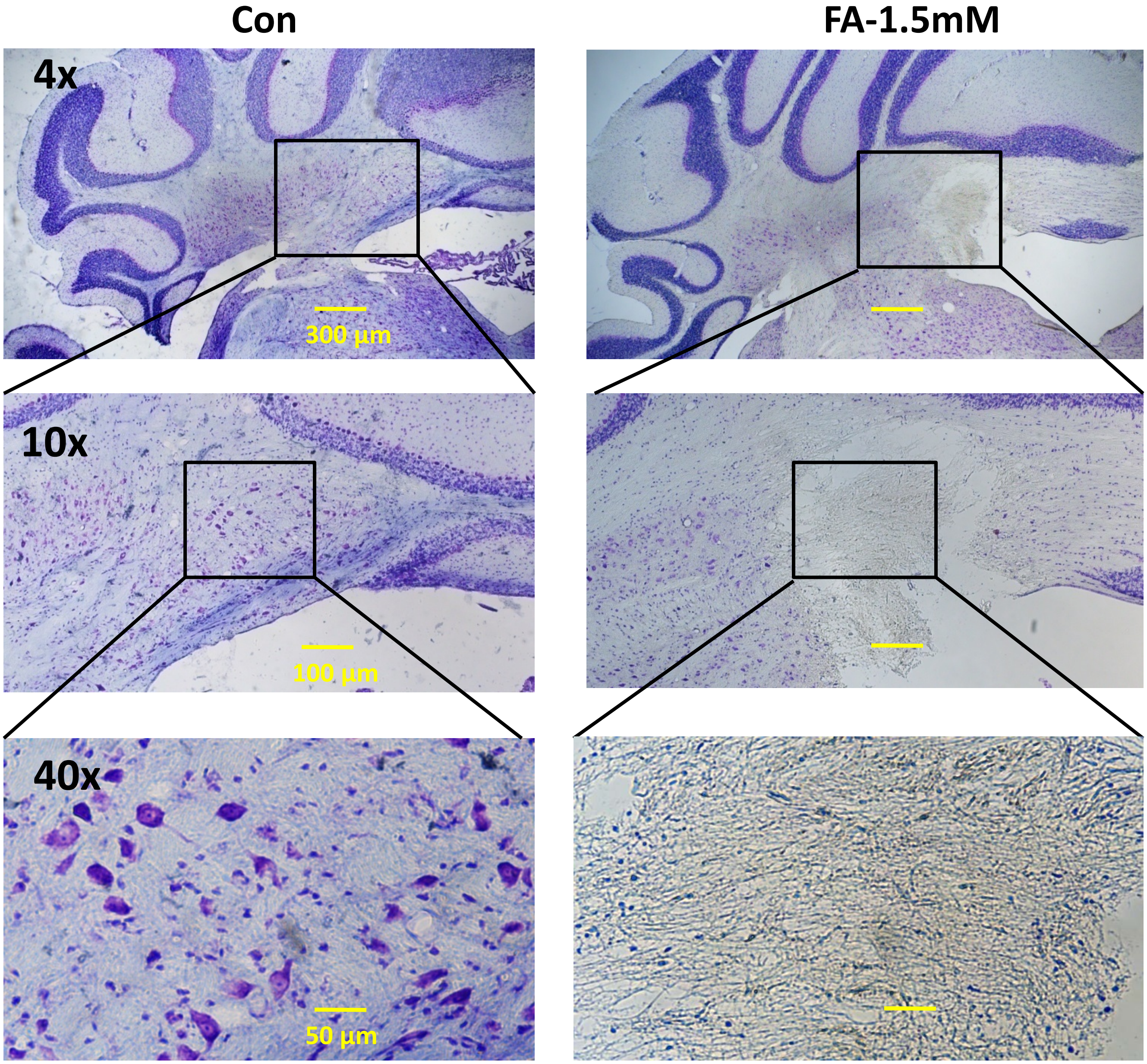

Fig. S11. Injection of formaldehyde at 1.5 mM into fastigial nucleus induced the death of cerebellar neurons stained by using Nissl staining solutions.

Supplementary Figure 12

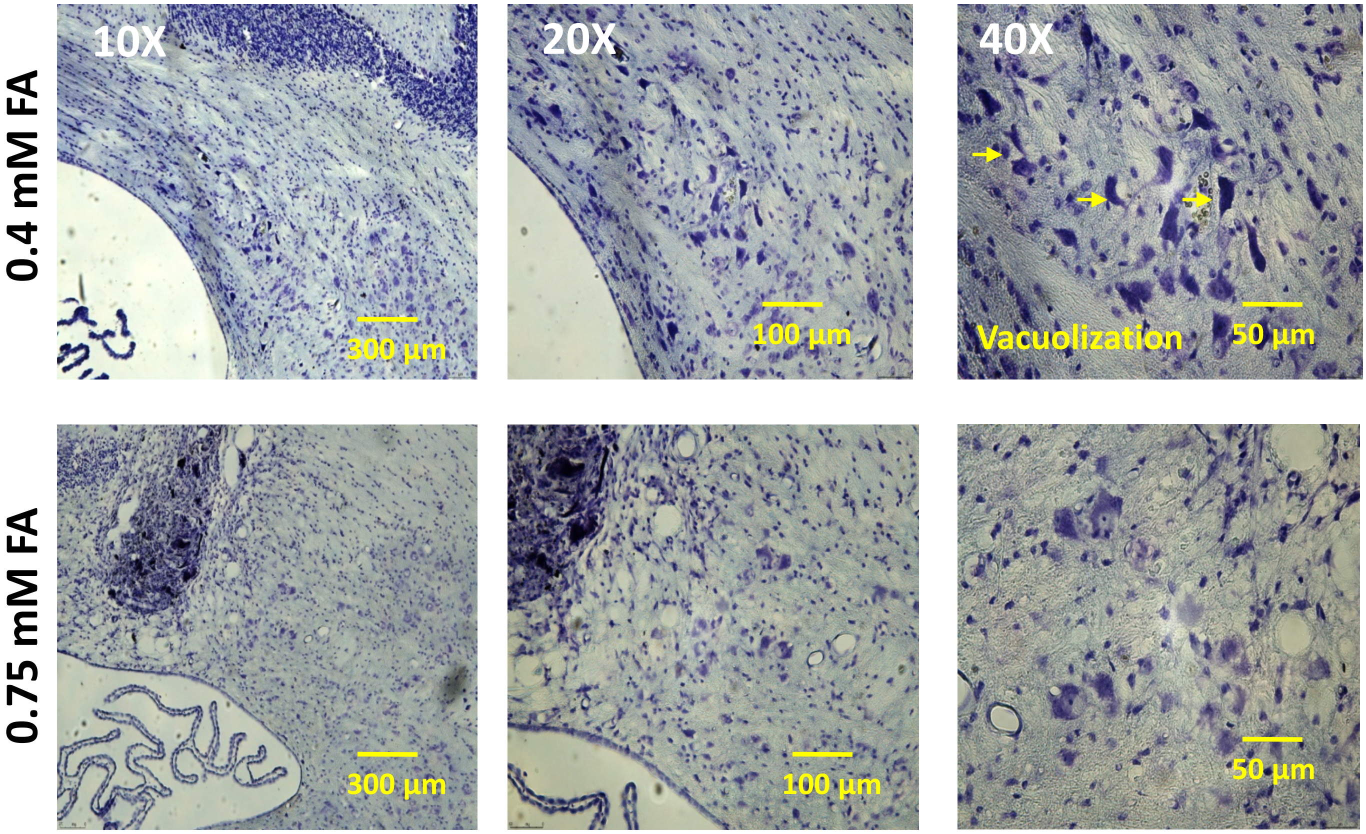

Fig. S12. Injection of formaldehyde at 0.4 and 0.75 mM into fastigial nucleus induced the death of cerebellar neurons stained by using Nissl staining solutions, respectively.

Supplementary Figure 13

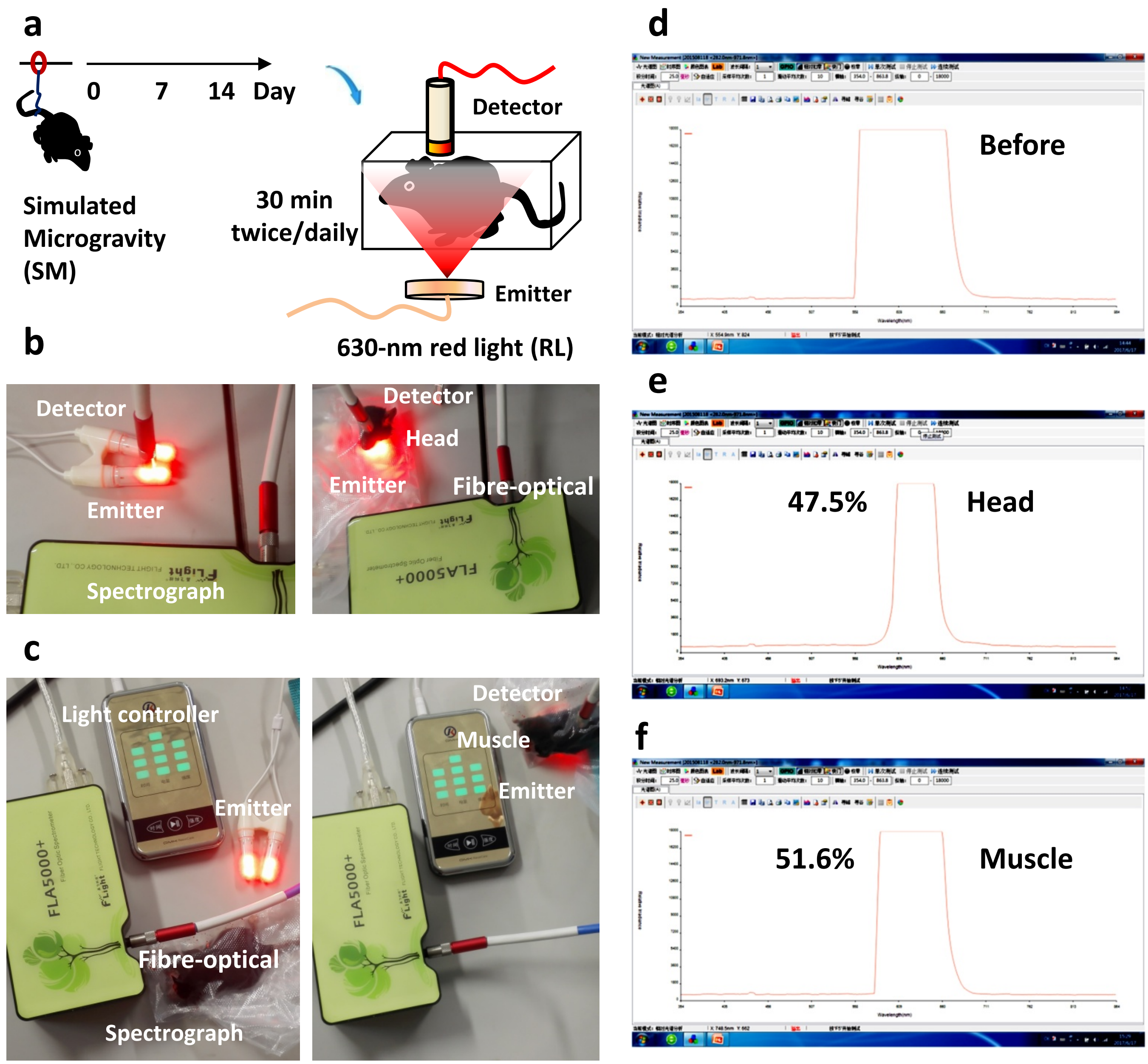

Fig. S13. The penetrate ratio of red light at 630 nm detected by spectrograph. (**a**) The free-moving SM mice illuminated in a box. (**b** and **c**) Detected the penetration ratio by spectrograph. (**d**) The light intensity of light emitter before penetrated into organism of mice. (**e** and **f**) Red light penetrated into the head and muscle with 47.5% and 51.6% penetration ratio, respectively.

Supplementary Figure 14

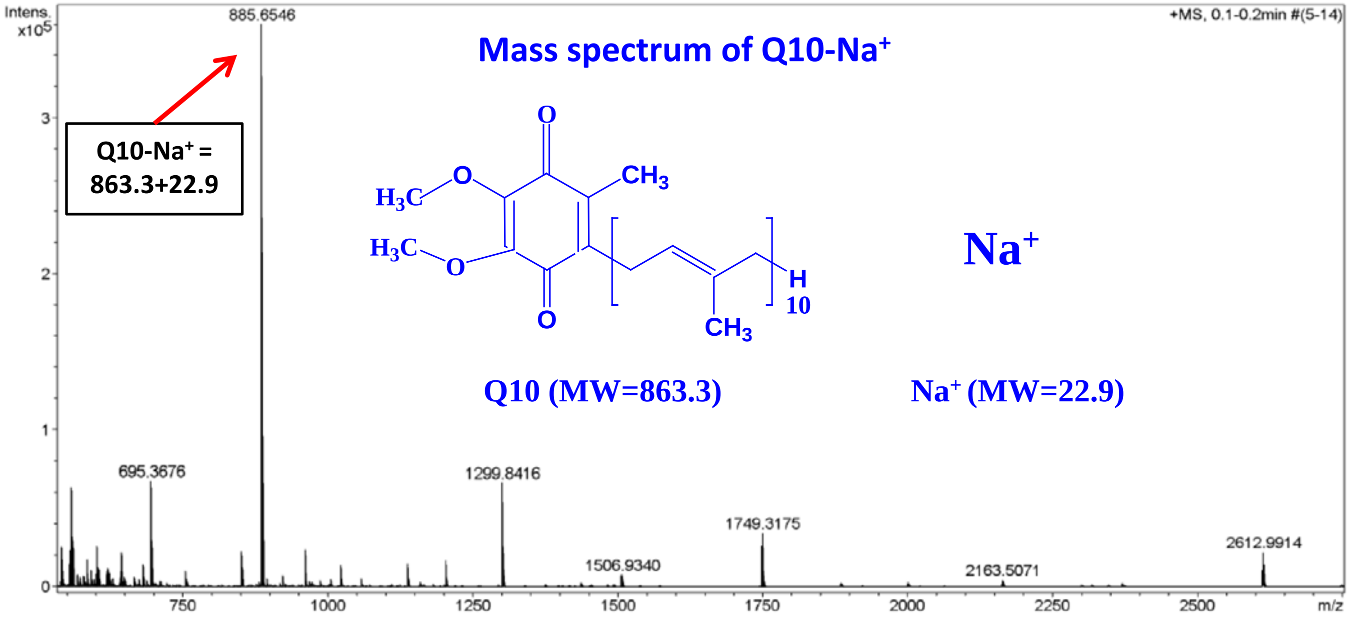

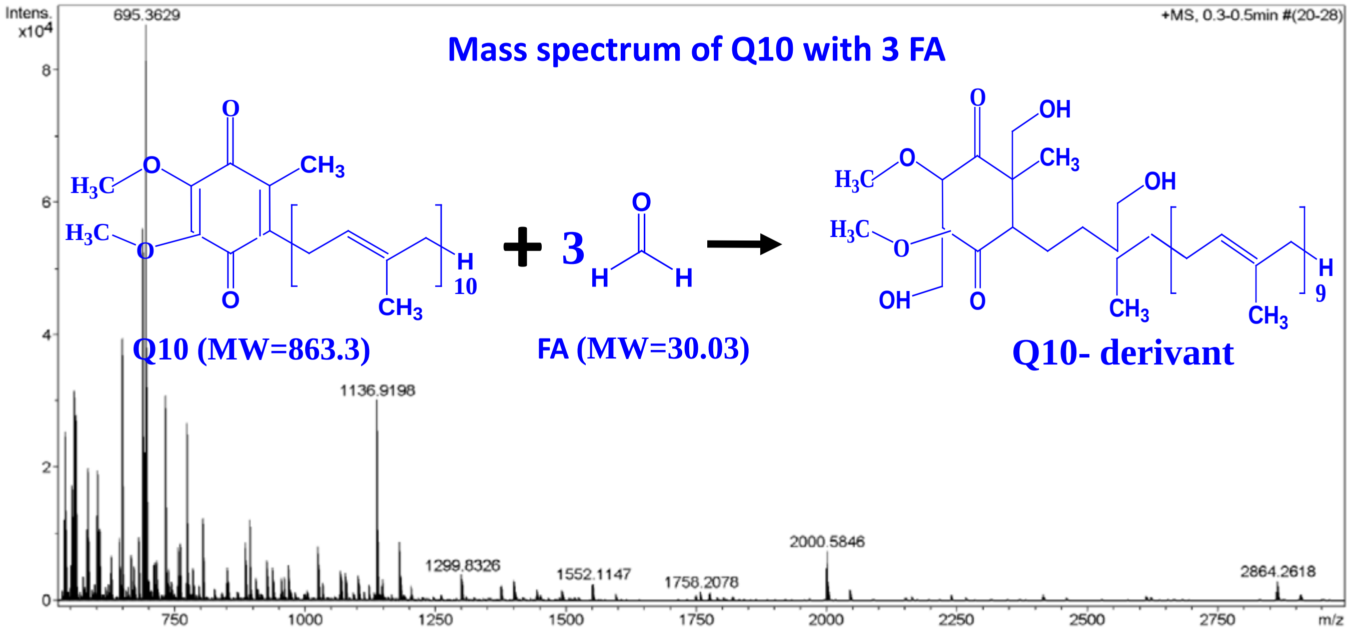

Fig S14. Q10 (100 μM) and FA (400 μM) were co-incubated in 100% alcohol solutions for 72 hours at 37 ℃, and the mixed solutions were examined by gas chromatography- tandem with mass spectrometry (GC-MS/MS) (#MicrOTOF-Q, Bruker, Germany)

Supplementary Figure 15

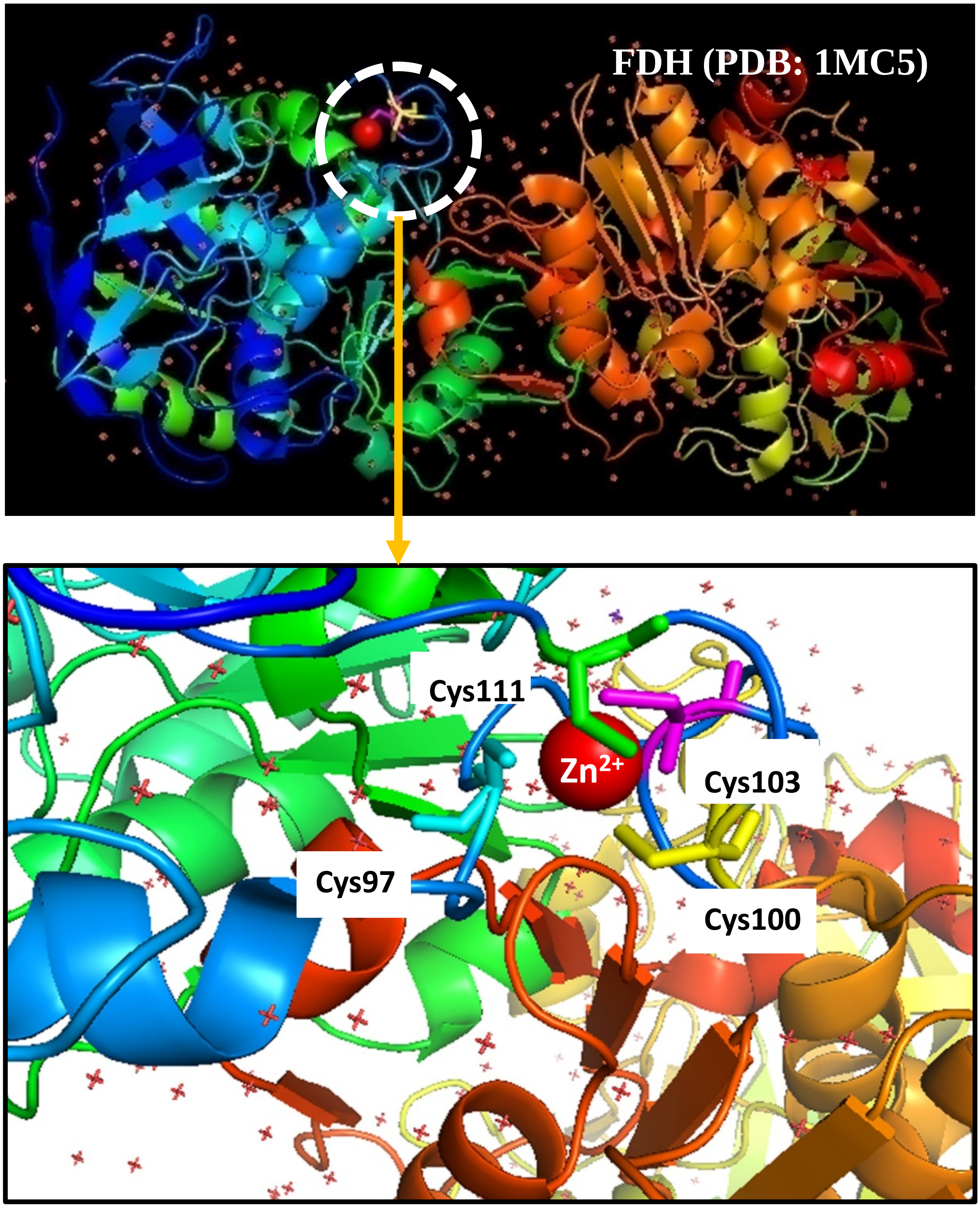

Fig. S15. The Cys 97 and 100 residues of FDH (PDB: 1MC5) were activated by 630-nm red light, which was analyzed by PyMOL software. Cys: cysteine. Cys111 (green); Cys103 (purple); Cys97 (dusty blue); Cys100 (yellow).

Supplementary Figure 16

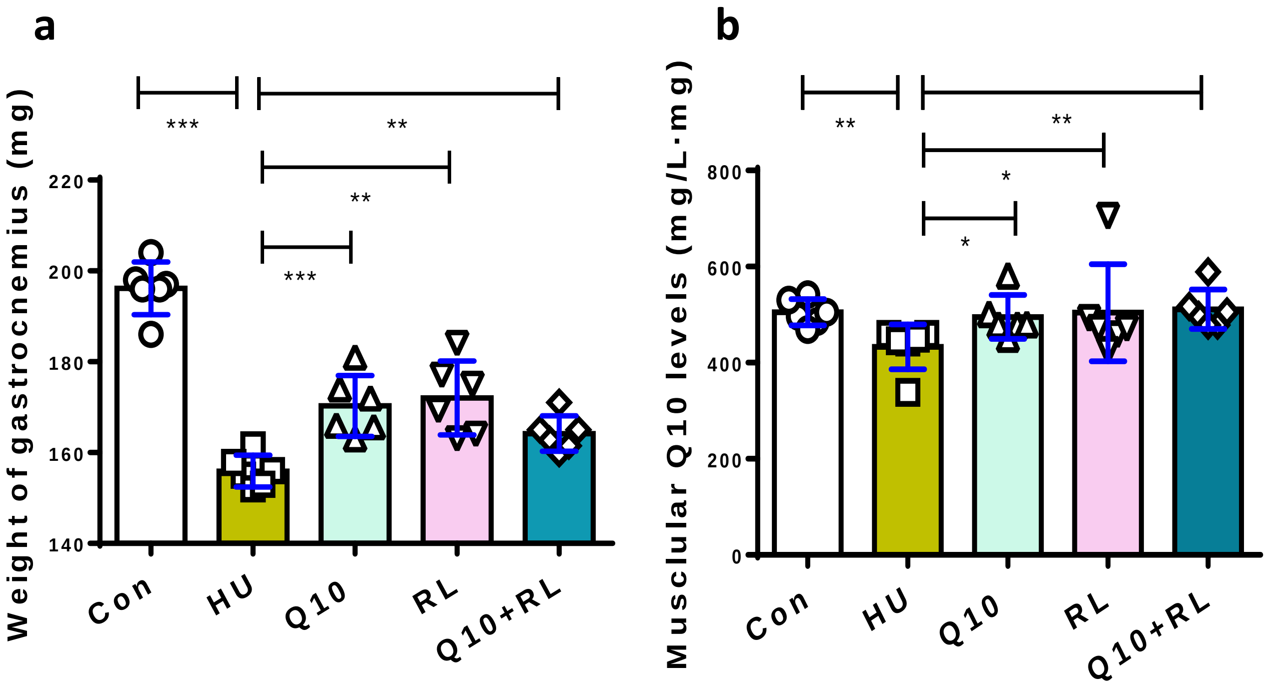

Fig. S16. Scavenging formaldehyde restored the weights of gastrocnemius and Q10 levels in HU mice. (**a**) The weight of gastrocnemius of these five groups' mice (n=6). HU: hindlimb unloading; Q10: HU mice intragastrically administered (i.g.) 30-nm coenzyme Q10; RL: HU mice illuminated with 630-nm red light. (**b**) Muscular Q10 levels detected by Q10 kits (n=6). Error bars show mean±S.E.M; *p<0.05; **p<0.01; ***p<0.001.

Supplementary Figure 17

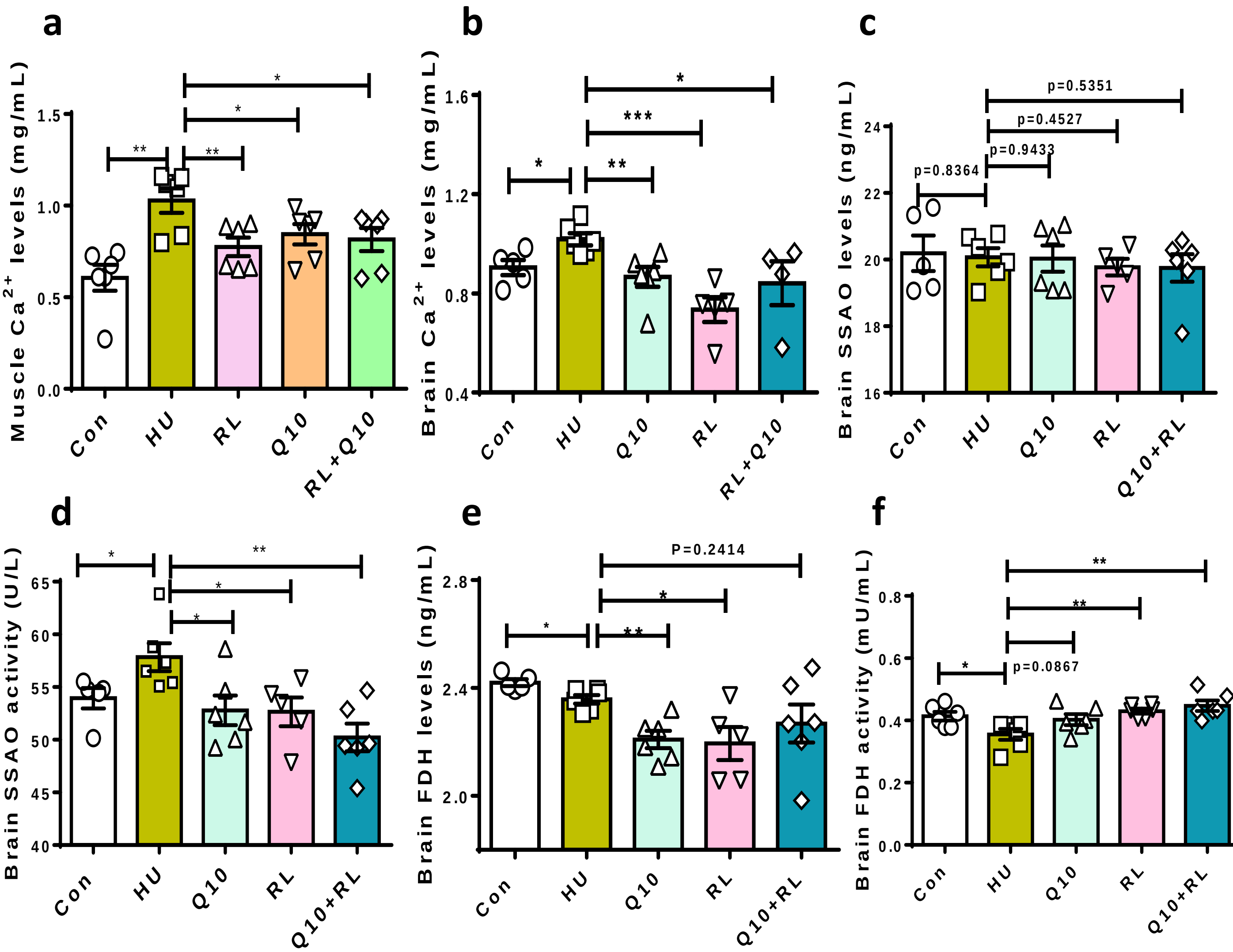

Fig. S17. The effects of the different treatments on the intracellular Ca^{2+}, SSAO and FDH in the muscles and brains of HU mice. (**a, b**) Changes in the Ca^{2+} contents in the muscles and brains. HU: hindlimb unloading. (**c, d**) Changes in the levels and activities of SSAO in the brains. (**e, f**) Changes in the levels and activities of FDH in the brains. Q10: HU mice intragastrically administered (i.g.) 30-nm coenzyme Q10; RL: HU mice illuminated with 630-nm red light. (n=6). Error bars show mean±S.E.M; *p<0.05; **p<0.01; ***p<0.001.

Supplementary Figure 18

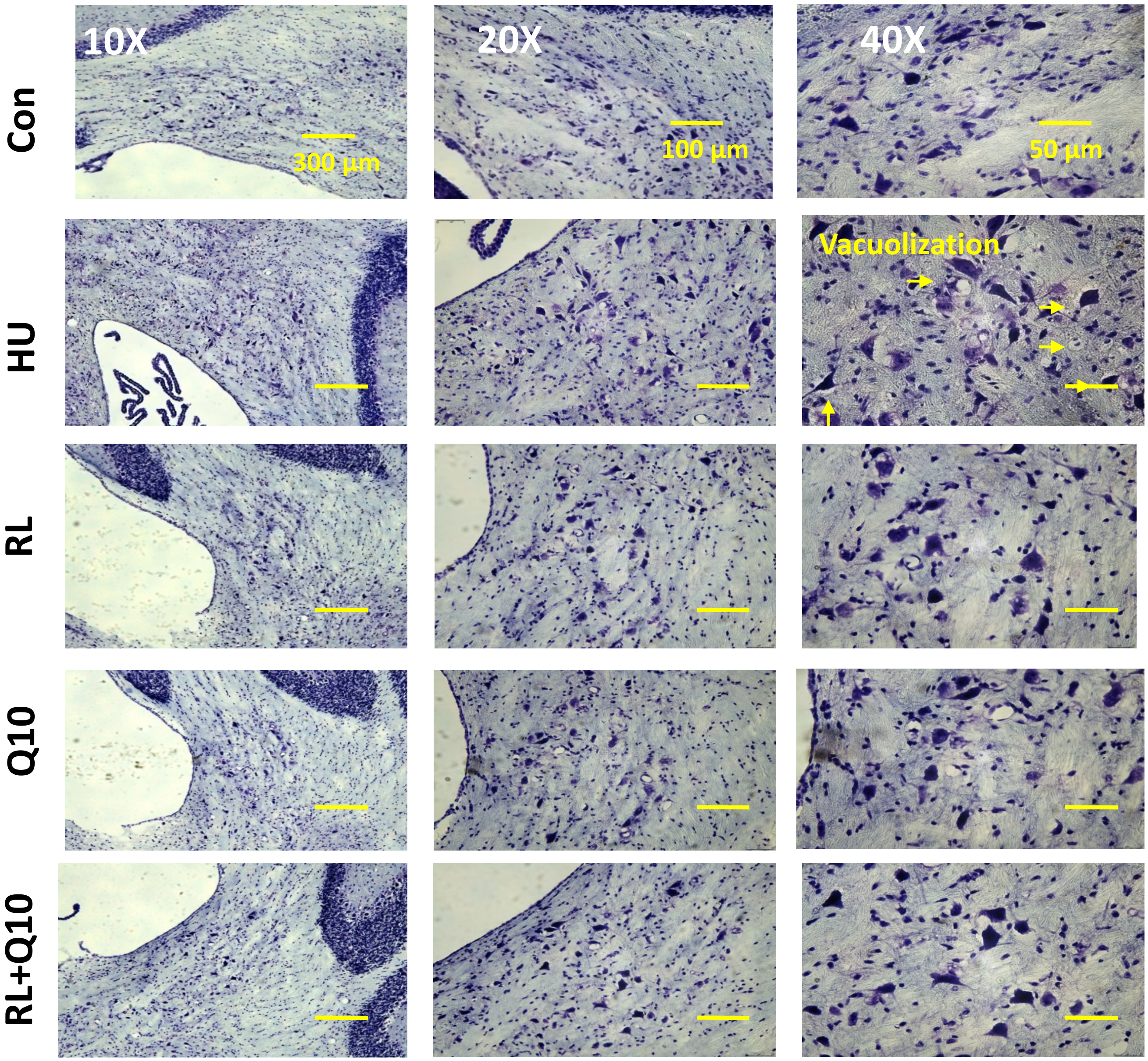

Fig. S18. The effects of the different treatments on the death of cerebellar neurons stained by using Nissl staining solutions. Con: control; HU: hindlimb unloading; RL: Red light; Q10: Nano-coenzyme Q10.

Supplementary Figure 19

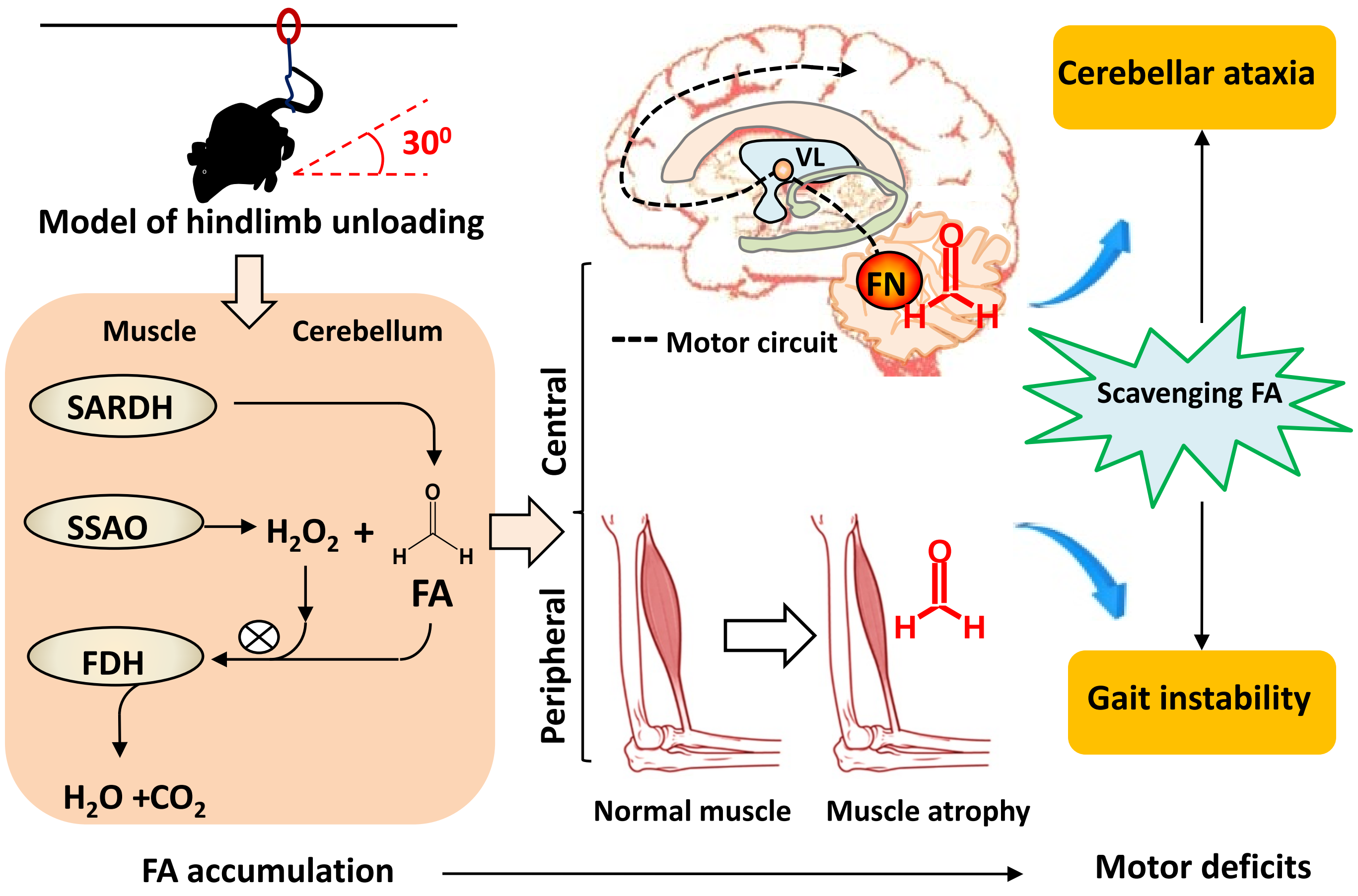

Fig S19. The model of scavenging HU-derived formaldehyde to rescue motor functions. FA: formaldehyde (HCHO); FDH: formaldehyde dehydrogenase; FN: fastigial nucleus; HU: hindlimb unloading; SARDH: sarcosine dehydrogenase; SSAO: semicarbazide-sensitive amine oxidase; VL: ventrolateral nucleus of the thalamus;.

Supplementary Figure 20

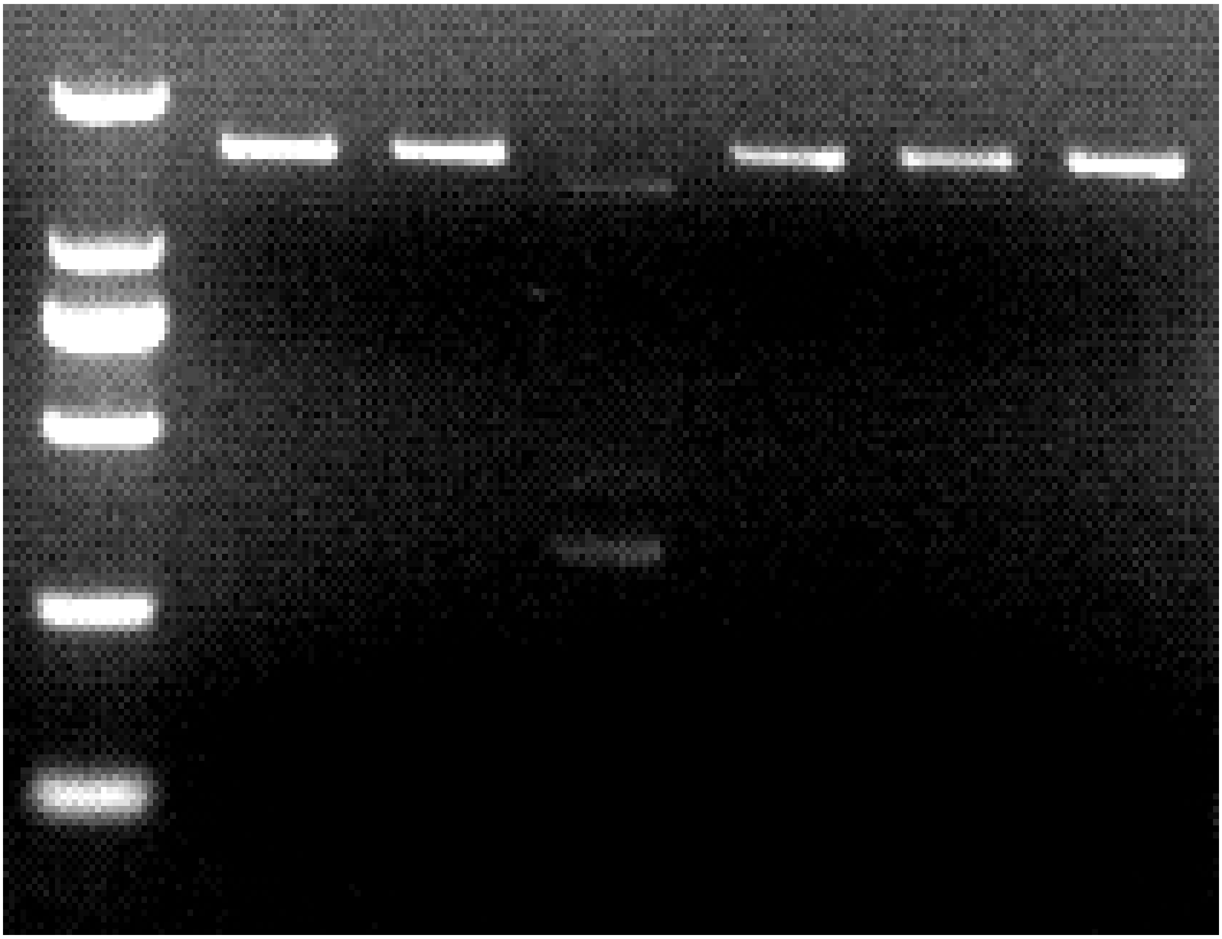

Fig S20. Uncropped blot from Figure 4
