# Supplementary material for: Accumulation of formaldehyde causes motor deficits in an in vivo model of hindlimb unloading
Source: Commun Biol. 2021 Aug 19;4:933. doi: 10.1038/s42003-021-02448-9 (PMC8376875; doi:10.1038/s42003-021-02448-9)
Supplement: Supplementary file 2 — Description of additional supplementary files [file 42003_2021_2448_MOESM2_ESM.pdf]

Description of Additional Supplementary Files

File Name: Supplementary Data 1

Description: Source data for all figures and supplementary figures.

File Name: Supplementary Movies 1-9

Description:

Supplementary Movie 1: Cell microgravity in the rotary cell culture system.

Supplementary Movie 2: Gait analysis.

Supplementary Movie 3: FN-injection of Formaldehyde-induced ataxia.

Supplementary Movie 4: Accelerating rotarod-Formaldehyde-injected group-Left 1,2,3; PBS-injected group-Right 4, 5.

Supplementary Movie 5: FN-injection of formaldehyde-Left-two mice with 1.5 mM; Right-3 mice with 0.75 mM.

Supplementary Movie 6: Beam walking-PBS-injected group.

Supplementary Movie 7: Beam walking-Formaldehyde-injected group.

Supplementary Movie 8: Bean walking-Injection of FA at 0.75mM.

Supplementary Movie 9: Bean walking-Injection of FA at 1.5mM.
